# HARDI-ZOOMit protocol improves specificity to microstructural changes in presymptomatic myelopathy

**DOI:** 10.1038/s41598-020-70297-3

**Published:** 2020-10-16

**Authors:** René Labounek, Jan Valošek, Tomáš Horák, Alena Svátková, Petr Bednařík, Lubomír Vojtíšek, Magda Horáková, Igor Nestrašil, Christophe Lenglet, Julien Cohen-Adad, Josef Bednařík, Petr Hluštík

**Affiliations:** 1 Department of Biomedical Engineering, University Hospital Olomouc, 779 00 Olomouc, Czech Republic; 2 Department of Neurology, Palacký University, 779 00 Olomouc, Czech Republic; 3 Division of Clinical Behavioral Neuroscience, Department of Pediatrics, University of Minnesota, Minneapolis, MN 55414 USA; 4 Central European Institute of Technology, Masaryk University, 625 00 Brno, Czech Republic; 5 Department of Neurology, University Hospital Brno, 625 00 Brno, Czech Republic; 6 Faculty of Medicine, Masaryk University, 625 00 Brno, Czech Republic; 7 Department of Medicine III, Clinical Division of Endocrinology and Metabolism, Medical University of Vienna, 1090 Vienna, Austria; 8 Department of Imaging Methods, Faculty of Medicine, University of Ostrava, 701 03 Ostrava, Czech Republic; 9 High Field MR Centre, Medical University of Vienna, Vienna, Austria; 10 Center for Magnetic Resonance Research, Department of Radiology, University of Minnesota, Minneapolis, MN 55414 USA; 11 Institute of Biomedical Engineering, Polytechnique Montreal, Montreal, Canada; 12 Department of Neurology, University Hospital Olomouc, 779 00 Olomouc, Czech Republic

**Keywords:** Data acquisition, Spine structure, Diagnostic markers, Spinal cord diseases, Biomedical engineering

## Abstract

Diffusion magnetic resonance imaging (dMRI) proved promising in patients with non-myelopathic degenerative cervical cord compression (NMDCCC), i.e., without clinically manifested myelopathy. Aim of the study is to present a fast multi-shell HARDI-ZOOMit dMRI protocol and validate its usability to detect microstructural myelopathy in NMDCCC patients. In 7 young healthy volunteers, 13 age-comparable healthy controls, 18 patients with mild NMDCCC and 15 patients with severe NMDCCC, the protocol provided higher signal-to-noise ratio, enhanced visualization of white/gray matter structures in microstructural maps, improved dMRI metric reproducibility, preserved sensitivity (SE = 87.88%) and increased specificity (SP = 92.31%) of control-patient group differences when compared to DTI-RESOLVE protocol (SE = 87.88%, SP = 76.92%). Of the 56 tested microstructural parameters, HARDI-ZOOMit yielded significant patient-control differences in 19 parameters, whereas in DTI-RESOLVE data, differences were observed in 10 parameters, with mostly lower robustness. Novel marker the white-gray matter diffusivity gradient demonstrated the highest separation. HARDI-ZOOMit protocol detected larger number of crossing fibers (5–15% of voxels) with physiologically plausible orientations than DTI-RESOLVE protocol (0–8% of voxels). Crossings were detected in areas of dorsal horns and anterior white commissure. HARDI-ZOOMit protocol proved to be a sensitive and practical tool for clinical quantitative spinal cord imaging.

## Introduction

After the Stejskal and Tanner 1965 experiment^1^, it took almost 3 decades until the diffusion magnetic resonance imaging (dMRI) was first applied to human brain imaging, utilizing estimation of the diffusion tensor imaging (DTI) model^2,3^. Since 2000 DTI has been used in the spinal cord (SC) imaging^4^ and become considered for clinical applications^5,6^, such as SC injury^7,8^, multiple sclerosis^4^, amyotrophic lateral sclerosis (ALS)^9^, Walerian degeneration^10^, adrenoleukodystrophy^11^ and degenerative cervical myelopathy (DCM)^12–18^. Recently, DTI detected microstructural SC injury^19^, and microstructural abnormality in patients with non-myelopathic degenerative cervical cord compression (NMDCCC) prior to development of subsequently expected neurological myelopathic symptoms and signs, i.e. DCM manifestation^20^. NMDCCC is a condition that is more frequent than previously estimated^20,21^. The SC compression at this stage may become an important target in the prediction and/or prevention of significant myelopathic clinical symptoms and signs mostly leading to disabling DCM^22^. The dMRI derived parameters (e.g. FA or MD) have distinguished healthy controls from patients with advanced DCM^12–14^, correlated with clinical disability in DCM patients^23^, and been able to detect signs of microstructural SC injury in NMDCCC patients in an exploratory study^20^. There is an urgent need to confirm the previous findings but also to develop and validate a dMRI protocol that is fast, reliable, clinically feasible, and yet possesses the ability to detect microstructural cervical cord injury in the NMDCCC stage with high sensitivity (SE) and specificity (SP).

Clinical dMRI protocols for the SC aim to acquire high spatial resolution data with good quality (SNR) over a clinically acceptable time period. Spatial resolution is often less than $1.25\times 1.25\times 5\,\hbox {mm}^3$ and short single-shell diffusion weighted protocols with 20–30 directions are almost exclusively employed^19^. Within each voxel, DTI fits a *seven* (6 parameters for the tensor + 1 parameter for signal intensity without diffusion weighting) *parameter single-compartment model* corresponding to an ellipsoid tensor oriented in one dominant direction. For the SC tissue, this tensor characterizes the average fiber bundle orientation within the voxel^3^.

One major limitation of such data is the inability to model complex intra-voxel fiber configurations (e.g. crossing fibers) due to a low angular resolution^24^. Yet, crossing fibers are known to be present in several areas of the SC at the histological level, e.g., the dorsal horns and the anterior commissure, where nerve fibers are oriented transversally, in contrast to the longitudinal organization of most of SC pathways. In dMRI data, crossing fibers are defined as multiple fiber bundles with different orientations within a single voxel, and have already been detected in the SC in animal models^25,26^ or humans^7,27,28^. Importantly, in a human study of injured SC the analysis of crossing fibers provided an increased specificity for various sub-types of white matter pathology^7^. Unfortunately, increasing angular resolution within current protocols would make them too long for clinical scanning.

The advanced clinical SC dMRI protocol should thus be fast, provide high angular resolution within acceptable scanning time and be complemented with an advanced crossing fiber model. For the first requirement, we have employed a reduced field-of-view (FOV) EPI sequence, e.g. syngo ZOOMit (*Siemens Medical, Erlangen, Germany*), which decreases the acquisition time without compromising dMRI data quality^29^. To achieve high angular resolution, we have designed a novel two-shell HARDI-ZOOMit^24,28,29^ protocol (high angular resolution diffusion imaging) covering the C3–C7 SC levels. Finally, to permit crossing fiber modeling, we have utilized the *three-compartment* “Ball and Stick and Stick model” that fits *eight parameters*^30^ and better characterizes dMRI data than the single-compartment DTI model^31^.

We have compared our new HARDI-ZOOMit protocol and model to a current standard clinical RESOLVE (REadout Segmentation Of Long Variable Echo trains) sequence^32^ that is being used and considered to provide high signal-to-noise ratio (SNR)^33^ in the evaluation of SC.

Multiple microstructural parameters were compared within SC regions of interest (ROIs), not only with single-subject mean/median and SD of dMRI metrics, but also advanced metrics such as skewness, kurtosis and several novel heuristic parameters. The sets of significant dMRI parameters and protocols’ SEs/SPs were evaluated with Wilcoxon rank-sum tests, analyses of covariance (ANCOVA), step-wise linear regressions and K-means clusterings. Moreover, SNR, mutual information between dMRI metric maps and SC anatomy, off-resonance susceptibility artifact effect and test–retest reproducibility were evaluated for each investigated dMRI protocol.

## Results

### Subject characteristics

The study sample consisted of a cohort of 33 NMDCCC patients (14 females, $56.7\pm 6.4\,\hbox {year}$), 13 age-comparable healthy controls (9 females, age $51.9\pm 9.4\,\hbox {year}$), and seven young healthy volunteers (3 females, age $27.4\pm 1.7\,\hbox {year}$; acquired twice for reproducibility evaluations). The radiological measurements, i.e. cross-sectional area (CSA) and compression ratio (CR), sub-divided the cohort of 33 NMDCCC patients to sub-group of 18 patients with mild NMDCCC (7 females, age $55.6\pm 6.1\,\hbox {year}$) and sub-group of 15 patients with severe NMDCCC (7 females, $58.1\pm 6.8\,\hbox {year}$). The neurological examination confirmed no DCM symptom presence for NMDCCC patients. A smaller proportion of NMDCCC cohort (13 out of 33 patients) displayed clinical symptoms and/or signs of cervical monoradiculopathy (mostly a radicular pain, less frequently a motor deficit in a corresponding myotome). Two-sample t-test identified the probability of age difference between age-comparable healthy controls and NMDCCC patients at $\hbox {p}=0.052$, and mild NMDCCC patients at $\hbox {p}=0.195$, and severe NMDCCC patients at $\hbox {p}=0.056$. To avoid the potential age or radiculopathy effects post-hoc ANCOVA or Wilcoxon rank-sum tests were performed for each presented significant between-group difference below.

### Acquisition protocols

**Figure 1:**
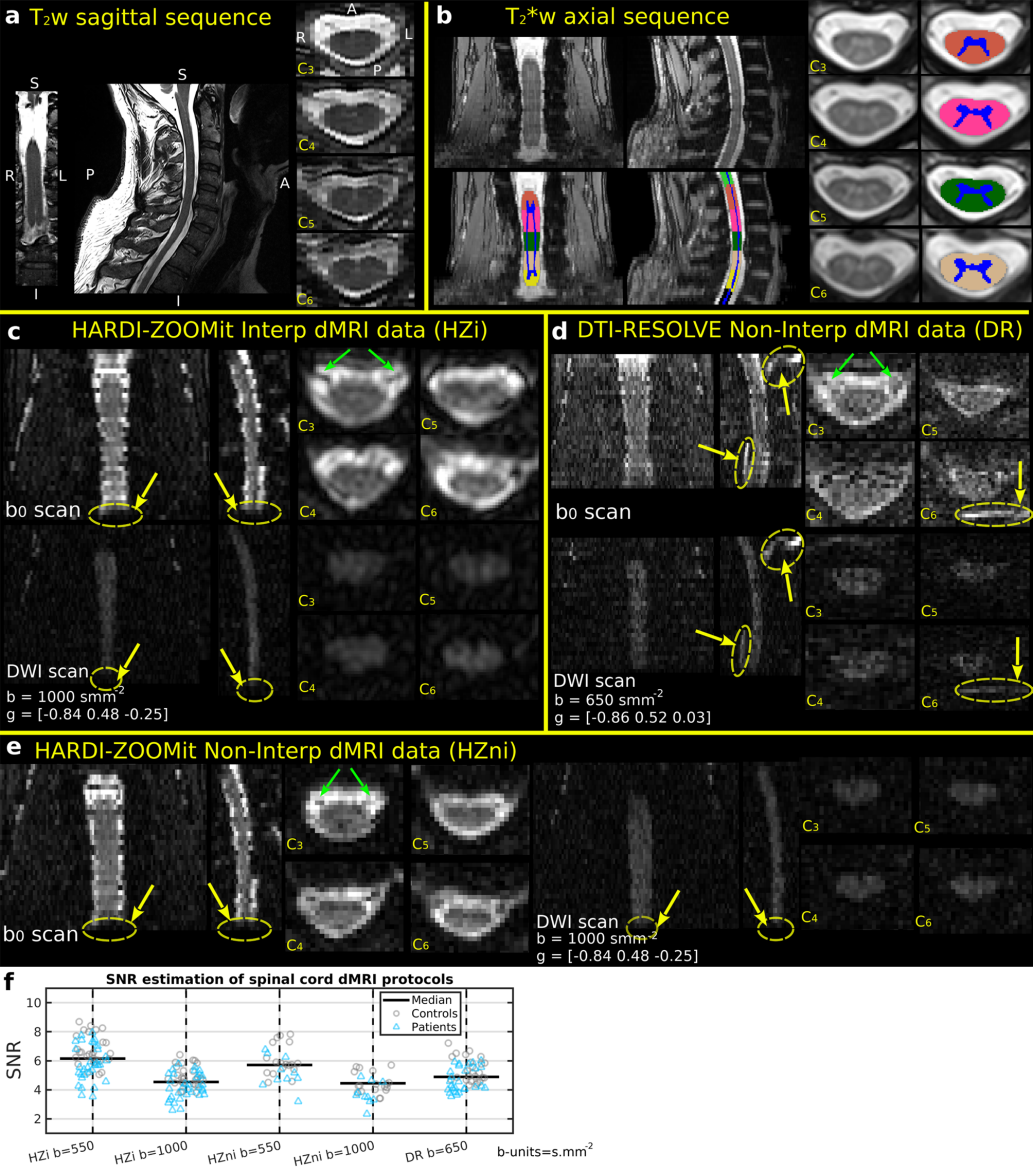
Raw anatomical MRI data, white and gray matter segmentation, and vertebral level labeling in a single-subject (**a**–**e**). Coronal (left), sagittal (middle) and axial (right) images with corresponding cervical (C) vertebral levels are shown; anatomical orientation: *S* superior, *I* inferior, *R* right, *L* left, *A* anterior, *P* posterior, as included in (**a**); anatomical plane order and direction orientation is the same in all other panels. (**a**), $\hbox {T}_2\hbox {w}$ sagittal sequence (**b**), $\hbox {T}_2^*\hbox {w}$ axial sequence with WM/GM segmentation and vertebral level labeling overlay (**c**). Diffusion non-weighted (i.e. $\hbox {b}_0$ scan in the first row of images) and weighted (i.e. DWI scan in the second row of images) images for the HARDI-ZOOMit protocol with Fourier domain interpolation during image reconstruction. Variable b (i.e. b-value) represents a magnitude of applied gradient waveform in the direction of a gradient vector **g**. Yellow arrows indicate areas of signal loss, green arrows the nerve roots in $\hbox {b}_0$ scan. (**d**) $\hbox {b}_0$ and DWI scans for the non-interpolated DTI-RESOLVE protocol. Order of $\hbox {b}_0$ and DWI scans and diffusion variable description same as in (**c**). Yellow arrows indicate locations of ghost artifacts, green arrows the nerve roots in $\hbox {b}_0$ scan. (**e**) $\hbox {b}_0$ (left) and DWI (right) scans for the non-interpolated HARDI-ZOOMit protocol. Diffusion variable description same as in (**c**). Yellow arrow indicate areas of signal loss, green arrows the nerve roots in $\hbox {b}_0$ scan. (**f**) Estimation of signal-to-noise ratio (SNR) in spinal cord DWI scans of investigated protocols.

A visualization of acquired MRI data with segmented and labeled white (WM) and gray (GM) matter in $\hbox {T}_2^*$-w axial space (i.e. T2TRA space) is presented in Fig. 1a–e. $\hbox {T}_2^*$-w axial scans provided a superior WM/GM contrast that was ideally suited for WM/GM segmentation (Fig. 1b). Both HARDI-ZOOMit protocols, interpolated and non-interpolated (Fig. 1c,e), were about 3 min 30 s faster when compared to the DTI-RESOLVE non-interpolated protocol (Fig. 1d). In comparison to DTI-RESOLVE protocol with b-value $650\,\hbox {s mm}^{-2}$ ($\hbox {SNR} \,5.1\pm 1.3$), HARDI-ZOOMit protocols generated diffusion weighted images with higher SNR level for b-value $550\,\hbox {s mm}^{-2}$ (interpolated $6.2\pm 1.2$, non-interpolated $5.7\pm 1.1$), and with lower SNR for b-value $1000\,\hbox {s mm}^{-2}$ (interpolated $4.5\pm 0.9$, non-interpolated $4.7\pm 0.9$), as anticipated. Full visualization of SNR results is shown in Fig. 1f. NMDCCC leads to a decrease in SNR for all protocols (Fig. 1f). A loss of SNR was also observed in both HARDI-ZOOMit protocols in the lower part of C7 vertebral level (Fig. 1c,e) and may be caused by the increased susceptibility artifact originating from the lungs. The used spherical two-shell HARDI acquisition scheme is shown in Fig. 4a.

### Diffusion MRI microstructural metrics

**Figure 2:**
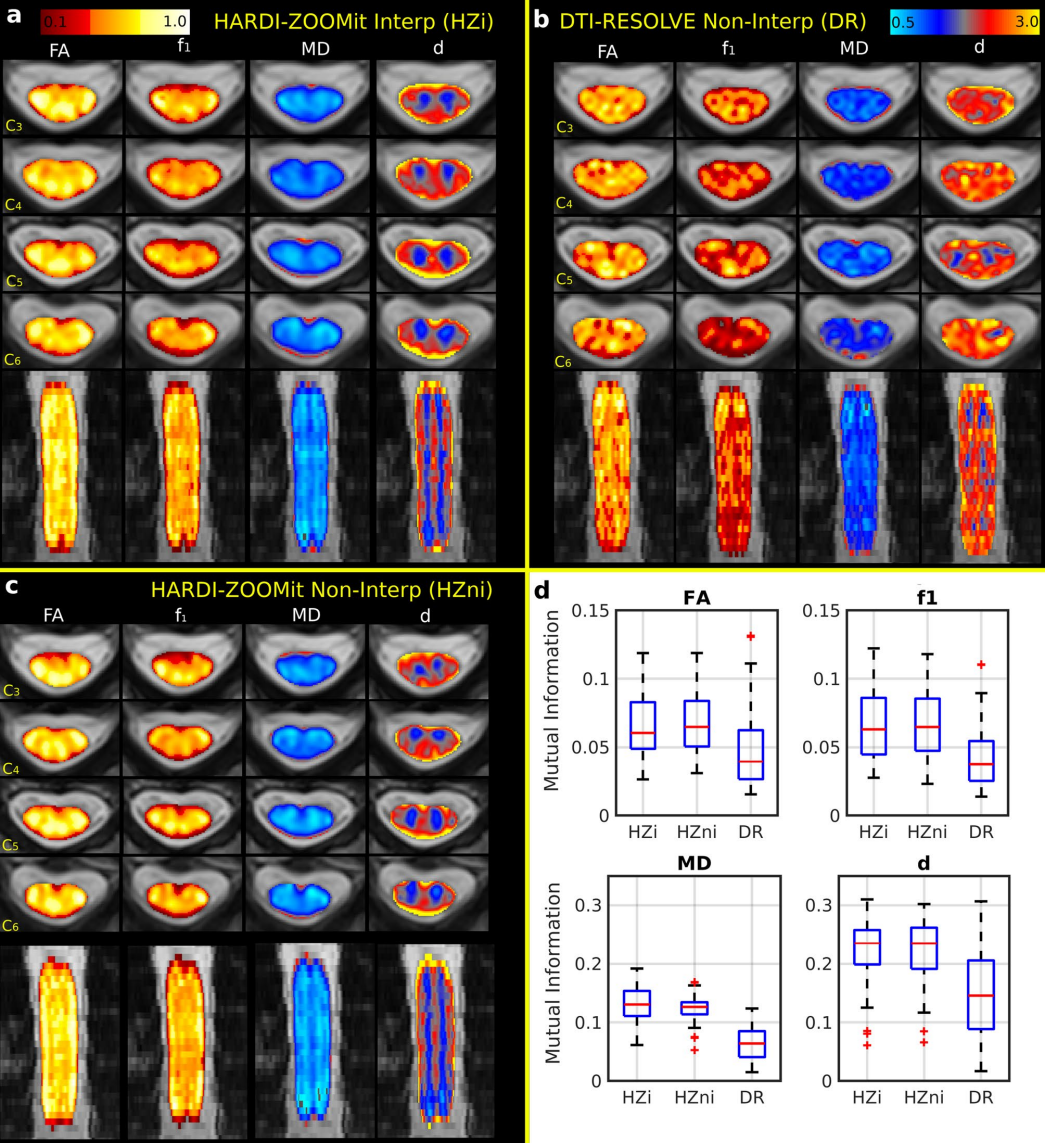
Diffusion MRI metrics and their correspondence to WM/GM structures in a single-subject (**a**–**c**) and across all participants (**d**). FA (DTI model fractional anisotropy), $f_1$ (Ball and Stick and Stick model partial volume of the 1^st^ principal diffusion direction), MD (DTI model mean diffusivity) and *d* (Ball and Stick and Stick model intra-voxel mean diffusivity) maps are shown for four representative axial slices and one coronal slice for the HARDI-ZOOMit interpolated protocol (**a**), the DTI-RESOLVE non-interpolated protocol (**b**) and the HARDI-ZOOMit non-interpolated protocol (**c**). All FA and $f_1$ maps use a “hot” colormap (i.e. red–orange–yellow–white colorbar on the top left panel). All MD and *d* maps are a “blue–yellow” colormap (i.e. lightblue–blue–gray–red–orange–yellow colorbar on the top right panel). All direction orientations of the axial (first four rows of images) or coronal slices (last row of slices) are the same as shown for the $\hbox {T}_2\hbox {w}$ sagittal sequence in Fig. 1a. (**d**) Distributions of mutual information between dMRI metrics and semi-threshold (i.e. background = 0, WM = 2 and GM = 1) WM/GM structure images demonstrate (in comparison to the DTI-RESOLVE protocol) increased mutual entropy level for both HARDI-ZOOMit protocols in all dMRI metrics. Ball and Stick and Stick model diffusivity map (*d*) has larger mutual entropy with WM/GM structures than DTI model MD map. Both diffusivity maps have larger mutual entropy with WM/GM structures than FA or $f_1$ maps. They still contain some information about WM/GM structures, while mutual information coefficients are > 0.

**Figure 3:**
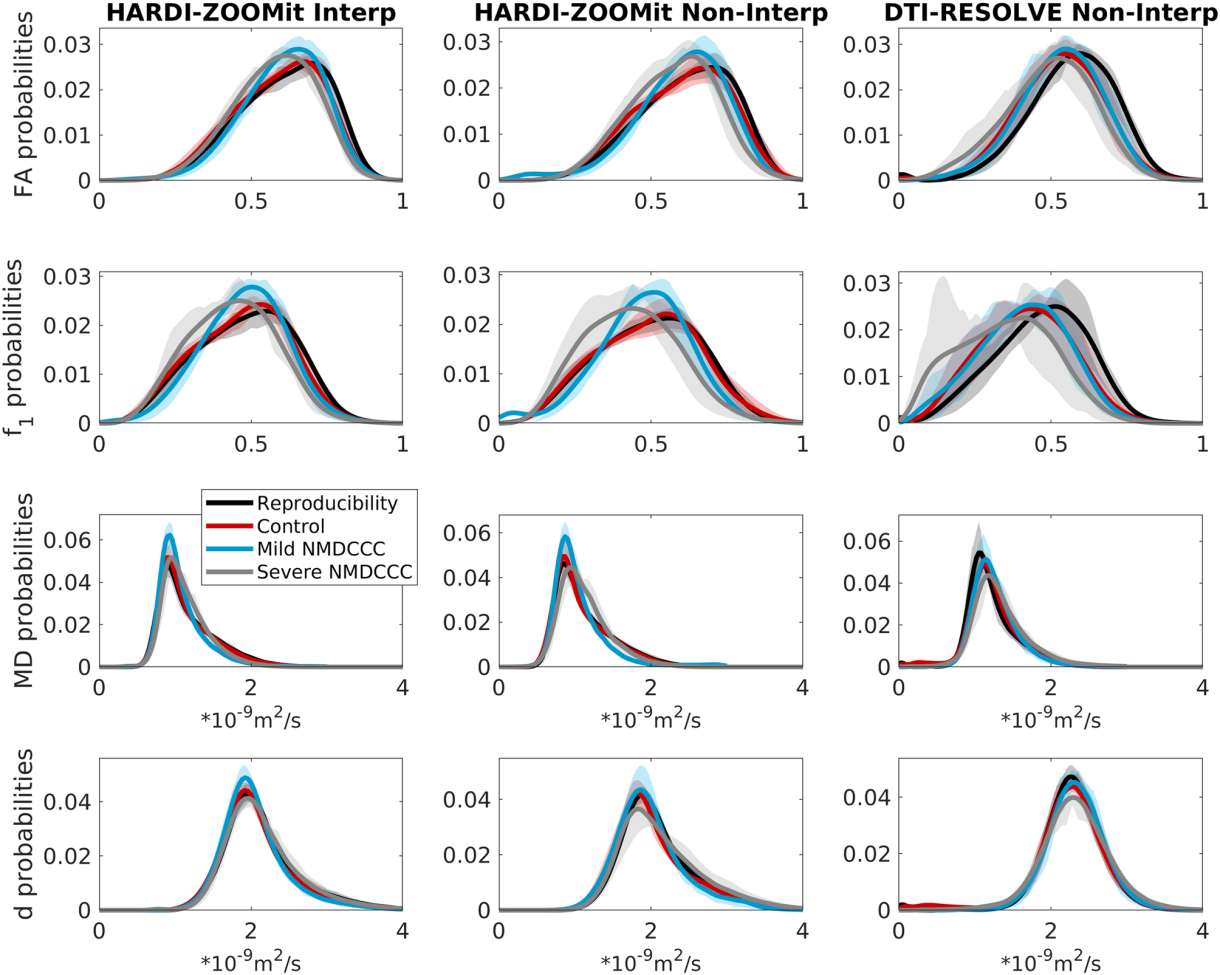
Group-averaged distributions of dMRI microstructural parameters from C3–C6 white matter. The confidence intervals (i.e. corresponding transparent colors) show Q1–Q3 quartiles. The intervals are narrow and distinct for most distributions, except $f_1$ for non-interpolated HARDI-ZOOMit protocol with lower number of participants, and except $f_1$ for non-interpolated DTI-RESOLVE protocol where precise $f_1$ and $f_2$ volume estimations might fail because of the protocol’s lower angular resolution. Trends in distributions are similar for both HARDI-ZOOMit protocols.

For dMRI analysis, the region of interest (ROI) was reduced to the C3–C6 vertebral levels due to the presence of artifacts at C7 level as described above. Clinical relevance should not be affected much since the expected C7 SC compression in DCM^34^ is presented only in 8–11% of cases in the used Caucasian middle-Europe population^21,35^. If the NMDCCC causes more extensive changes even above the affected C7 segment, C3–C6 ROI might still work fine. Diffusion MRI maps characterizing the SC microstructure, warped into T2TRA space, are shown in Fig. 2a–c. As expected, fractional anisotropy (FA) and partial volume of the primary fiber bundle direction ($f_1$) were lower in GM and higher in the WM corresponding to lateral corticospinal tracts and dorsal columns (i.e. gracile and cuneate fasciculi) for both HARDI-ZOOMit protocols (Fig. 2a,c). In contrast, GM/WM difference was more difficult to identify in the DTI-RESOLVE Non-Interp FA and $f_1$ maps (Fig. 2b). Single-subject mean/median values of FA and $f_1$ were higher in the HARDI-ZOOMit protocols than in the DTI-RESOLVE protocol for both WM (Fig. 3, Supplementary Figs. S1, S2) and GM (Supplementary Figs. S1–S3) structures.

Mean diffusivity maps (i.e. MD from the DTI model and *d* from the Ball and Stick and Stick model) displayed higher values at the edge of SC over all protocols (Fig. 2a–c). The spatial definition of the WM/GM boundary was clearer in HARDI-ZOOMit protocols than DTI-RESOLVE protocol (Fig. 2a,c). Over all dMRI protocols, DTI estimated lower MD values than Ball and Stick and Stick model for *d* (Figs. 2a–c, 3, Supplementary Figs. S3–S5). HARDI-ZOOMit protocols increased the mutual information (i.e. a usable similarity measure of degree of joint entropy in two multi-modal images with a non-linear contrast transform function, Eq. (7); see more information in Methods) between dMRI metric maps and semi-binarized images (i.e. $\hbox {background} = 0$, $\hbox {WM} = 2$ and $\hbox {GM} = 1$) of WM/GM structures (Fig. 2d).

Most distributions of dMRI parameters estimated from WM revealed narrow confidence intervals for each group of subjects (Fig. 3). These distributions also showed distinct properties between patients and healthy controls in the HARDI-ZOOMit protocol (Fig. 3). DTI-RESOLVE non-interpolated (possibly due to lower angular resolution) has higher inter-subject variance in confidence intervals for $f_1$ (Fig. 3). Results from GM demonstrated higher between-group overlap (Supplementary Fig. S3), except MD and *d* estimated from DTI-RESOLVE showing distinct properties between patients and healthy controls (Supplementary Fig. S3).

**Figure 4:**
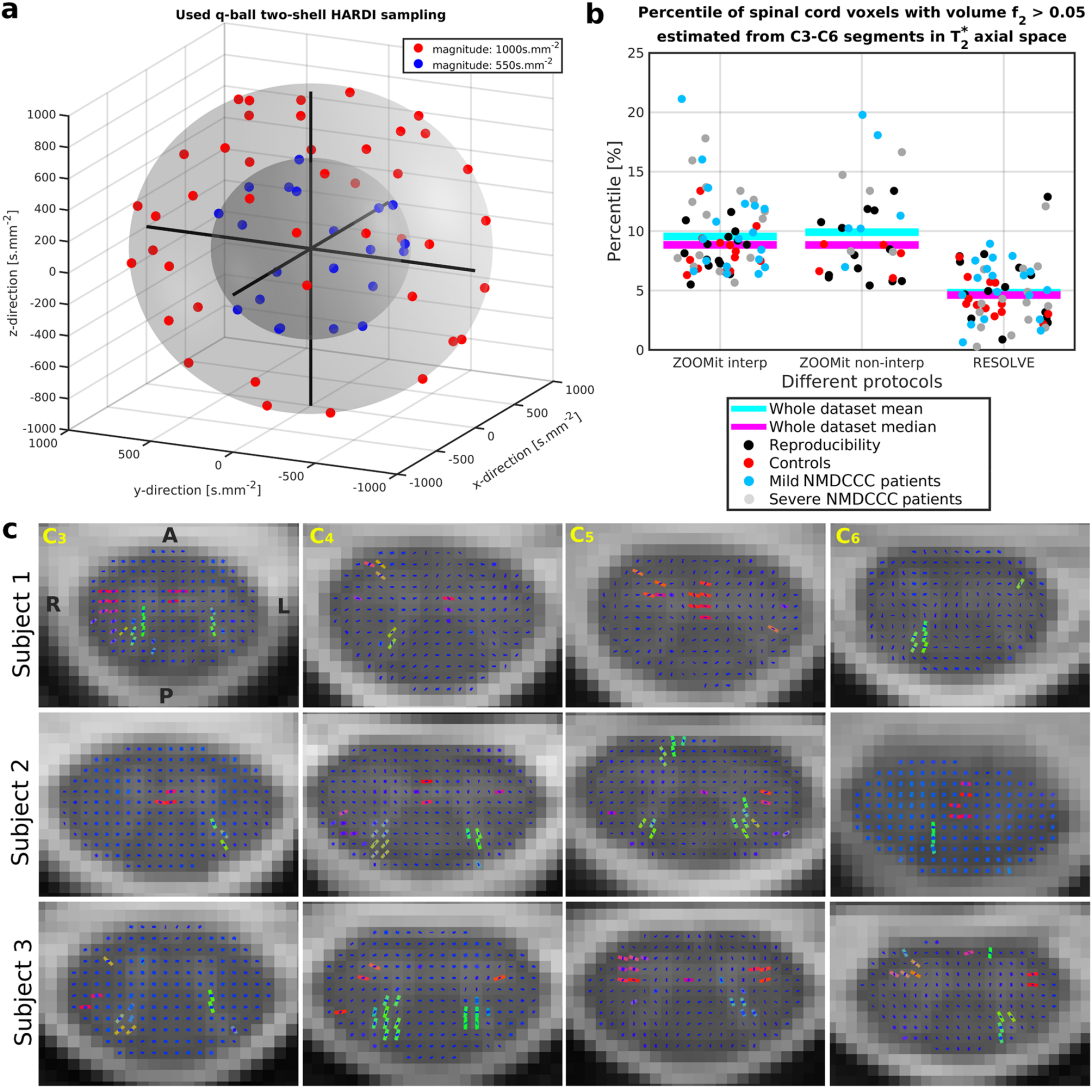
HARDI acquisition scheme and detected crossing fibers. (**a**) The graph visualizes 63 unique MR field gradient directions uniformly sampled over two spherical shells of the q-space. Caruyer et al.^57^ sampler was used to design the HARDI acquisition scheme. (**b**) Voxels with significant 2^nd^ fiber orientations (i.e. crossing fibers) were detected for all dMRI protocols. No significant difference in voxel numbers was found between groups. (**c**) As shown for results of HARDI-ZOOMit Interp protocol in native diffusion space, detected crossing fibers are mostly located near the dorsal horns and in the anterior white commissure. Visualizations are shown for three representative participants with four selected C3–C6 axial slices. Orientations are the same over all presented slices with *A* anterior, *P* posterior, *L*left, *R* right directions. The orientation of detected fiber bundles are visualized as RGB (red–green–blue) color-coded lines (i.e. red—right–left direction, green—anterior–posterior direction, blue—superior–inferior direction). The blue dot in the middle of each SC voxel demonstrates an axial projection of the intra-voxel primary fiber bundle with a major superior–inferior direction. In voxels where projections of two lines are present, crossing fibers were detected.

HARDI acquisition protocols enable to model and visualize multiple fiber bundles within single voxel, i.e., intra-voxel crossing fiber conformations^24,36^. All protocols identified 2^nd^ fiber bundle directions as the 2^nd^ significant fully anisotropic Stick^30^ compartment (i.e. $f_2>0.05$) especially near to the dorsal horns. HARDI-ZOOMit protocols estimated a higher number of crossing fiber configurations compared to DTI-RESOLVE protocol (Fig. 4b), likely due to the higher angular resolution afforded by HARDI-ZOOMit protocols. Crossing fibers were particularly detected in areas of dorsal horns and anterior white commissure (Fig. 4c). Apart from our own preliminary data^28^, this presents one of the first visualizations of crossing fibers from contiguous SC human in-vivo dMRI data (Fig. 4c). However, the amount of detected crossings (Fig. 4b,c) is possibly still underestimated^25–27^. Still, the intra-voxel modelling of crossings yielded better local underlying microstructural decomposition providing maps more close to WM/GM structures (e.g. $f_1$ vs FA and *d* vs MD in Fig. 2d) and higher separation of $f_1$-based dMRI metrics in comparison to complementary FA-based metrics (Table 1). Significant local partial volumes $f_2$ (i.e. $f_2>0.05$) of the 2^nd^ crossing fiber bundles possibly filter out from $f_1$ maps a variability portion which makes local FA values falsely lower and appearing as flatter tensor of the underlying microstructure for the major fiber bundle than the ground truth is.

**Table 1:** dMRI metrics with significant differences between age-comparable healthy controls and NMDCCC patients with mild or severe compression.

Parameter	Shortcut	HARDI-ZOOMit Interp.							DTI-RESOLVE non-Interp.						
		Control	Mild Comp. patients			Severe Comp. patients			Control	Mild Comp. patients			Severe Comp. patients		
		Median	Median	diff %	p value	Median	diff %	p value	Median	Median	diff %	p value	Median	diff %	p value
FA_WM_STD	FAwS	0.1422	0.1286	− 9.60	**6.10E−3**	0.1356	− 4.65	5.30E−2	0.1266	0.1239	− 2.11	4.12E−1	0.1325	4.72	1.00E+1
FA_WM_kurtosis	FAwK	2.4934	2.8241	13.26	**5.35E−4**	2.6698	7.07	1.66E−2	2.7666	2.8592	3.35	8.89E−1	2.7658	− 0.03	5.19E−1
f1_WM_STD	f1wS	0.1512	0.1323	− 12.55	**6.10E−3**	0.1397	− 7.61	1.29E−2	0.1398	0.1318	− 5.71	2.54E−1	0.1304	− 6.75	2.49E−1
f1_WM_skewness	f1wSK	− 0.1315	− 0.0805	− 38.82	6.74E−1	0.0313	− 123	**6.57E−3**	− 0.0685	− 0.0078	− 88.68	5.89E−1	0.1390	− 303	1.67E−1
f1_WM_kurtosis	f1wK	2.4683	2.7209	10.23	**1.82E−4**	2.7330	10.72	**3.20E−3**	2.6254	2.6115	− 0.53	6.74E−1	2.7310	4.02	8.90E−1
MD_WM_median	MDwM	1.0509	0.9951	− 5.31	**5.40E−3**	1.0578	0.65	4.61E−1	1.1614	1.1860	2.12	5.62E−1	1.2524	7.83	1.29E−2
MD_WM_mean	MDwm	1.1545	1.0576	− 8.39	**2.51E−3**	1.1005	− 4.68	1.97E−1	1.2414	1.2345	− 0.56	7.64E−1	1.3230	6.58	2.40E−2
MD_WM_STD	MDwS	0.3561	0.2621	− 26.39	**1.10E−3**	0.2863	− 19.60	**2.28E−4**	0.3069	0.2708	− 11.76	3.56E−2	0.3150	2.64	9.63E−1
d_WM_STD	dwS	0.4893	0.4325	− 11.62	**4.77E−3**	0.4545	− 7.11	2.69E−1	0.3727	0.3544	− 4.89	1.93E−1	0.4238	13.72	7.12E−1
d_WM_skewness	dwSK	1.1506	1.1119	− 3.36	9.20E−1	0.9342	− 18.80	**6.57E−3**	0.1457	0.2125	45.86	4.35E−1	0.2112	44.99	3.57E−1
MD_GM_median	MDgm	0.8300	0.8406	1.28	2.38E−1	0.8933	7.64	**4.95E−3**	1.0577	1.1145	5.37	4.75E−2	1.1772	11.29	**4.95E−3**
MD_GM_mean	MDgm	0.8573	0.8633	0.70	4.35E−1	0.9048	5.53	1.46E−2	1.0657	1.1255	5.61	2.63E−2	1.1975	12.37	**2.03E−3**
MD_GM_skewness	MDgSK	1.6110	1.4599	− 9.38	6.17E−1	1.2434	− 22.81	5.89E−2	0.0600	0.5163	760	**7.76E−3**	0.6187	931	**1.07E−3**
d_GM_median	dgM	1.4979	1.5211	1.55	5.22E−2	1.6111	7.56	**6.57E−3**	1.9277	1.9717	2.29	2.63E−2	2.0262	5.11	**1.26E−3**
d_GM_mean	dgm	1.5401	1.5595	1.26	3.92E−2	1.6498	7.13	**2.75E−3**	1.8827	1.9816	5.26	1.54E−2	2.0449	8.62	**5.50E−4**
MD_WM-GM_median	MDwgM	0.2122	0.1470	− 30.71	**8.04E−5**	0.1711	− 19.35	1.88E−2	0.1185	0.0913	− 23.00	9.82E−3	0.1032	− 12.94	2.14E−1
MD_WM-GM_mean	MDwgm	0.2803	0.1906	− 32.00	**6.80E−5**	0.1990	− 29.00	**1.90E−4**	0.1676	0.1172	− 30.09	**1.27E−3**	0.1244	− 25.82	3.41E−2
d_WM-GM_median	dwgM	0.5129	0.4339	− 15.41	**1.32E−4**	0.4122	− 19.63	**1.48E−3**	0.3864	0.3446	− 10.82	4.32E−2	0.2546	− 34.11	**4.29E−3**
d_WG-GM_mean	dwgm	0.5676	0.4580	− 19.31	**2.41E−5**	0.4321	− 23.87	**2.28E−4**	0.3823	0.3340	− 12.64	5.72E−2	0.2373	− 37.92	**3.71E−3**
FA_WM_heuristic	FAwH	0.4729	0.5076	7.36	2.63E−2	0.5349	13.13	**6.52E−4**	0.5467	0.5569	1.87	6.74E−1	0.5412	− 1.00	6.45E−1
f1_WM_heuristic	f1wH	0.5362	0.5819	8.52	**4.21E−3**	0.5897	9.98	**4.29E−3**	0.5789	0.5941	2.64	7.64E−1	0.5505	− 4.90	4.07E−1
MD_WM_heuristic	MDwH	0.5683	0.6706	17.99	**7.18E−4**	0.6403	12.67	**3.20E−3**	0.5432	0.5755	5.94	4.84E−1	0.4757	− 12.42	6.54E−2
d_WM_heuristic	dwH	0.5002	0.5513	10.21	**6.10E−3**	0.5023	0.43	9.27E−1	0.3799	0.3538	− 6.86	4.12E−1	0.3411	− 10.22	3.11E−1
MD_GM_heuristic	MDgH	0.4956	0.5400	8.96	1.05E−1	0.5892	18.90	**1.48E−3**	0.7732	0.7345	− 5.00	4.59E−1	0.6435	− 16.78	**4.95E−3**
d_GM_heuristic	dgH	0.4872	0.4550	− 6.60	9.66E−2	0.3537	− 27.41	**5.71E−3**	0.0774	0.0533	− 31.08	5.22E−2	0.0527	− 31.82	1.53E−1

List of parameters with their corresponding labels as used in Fig. 6 along with statistics and Wilcoxon rank-sum test *p* values for HARDI-ZOOMit Interp and DTI-RESOLVE Non-Interp protocols. Significant *p* values ($p_{FWE}<0.05\approx 8.33\hbox {E}{-}3$) are highlighted in bold.

### Descriptive statistics parameters

**Figure 5:**
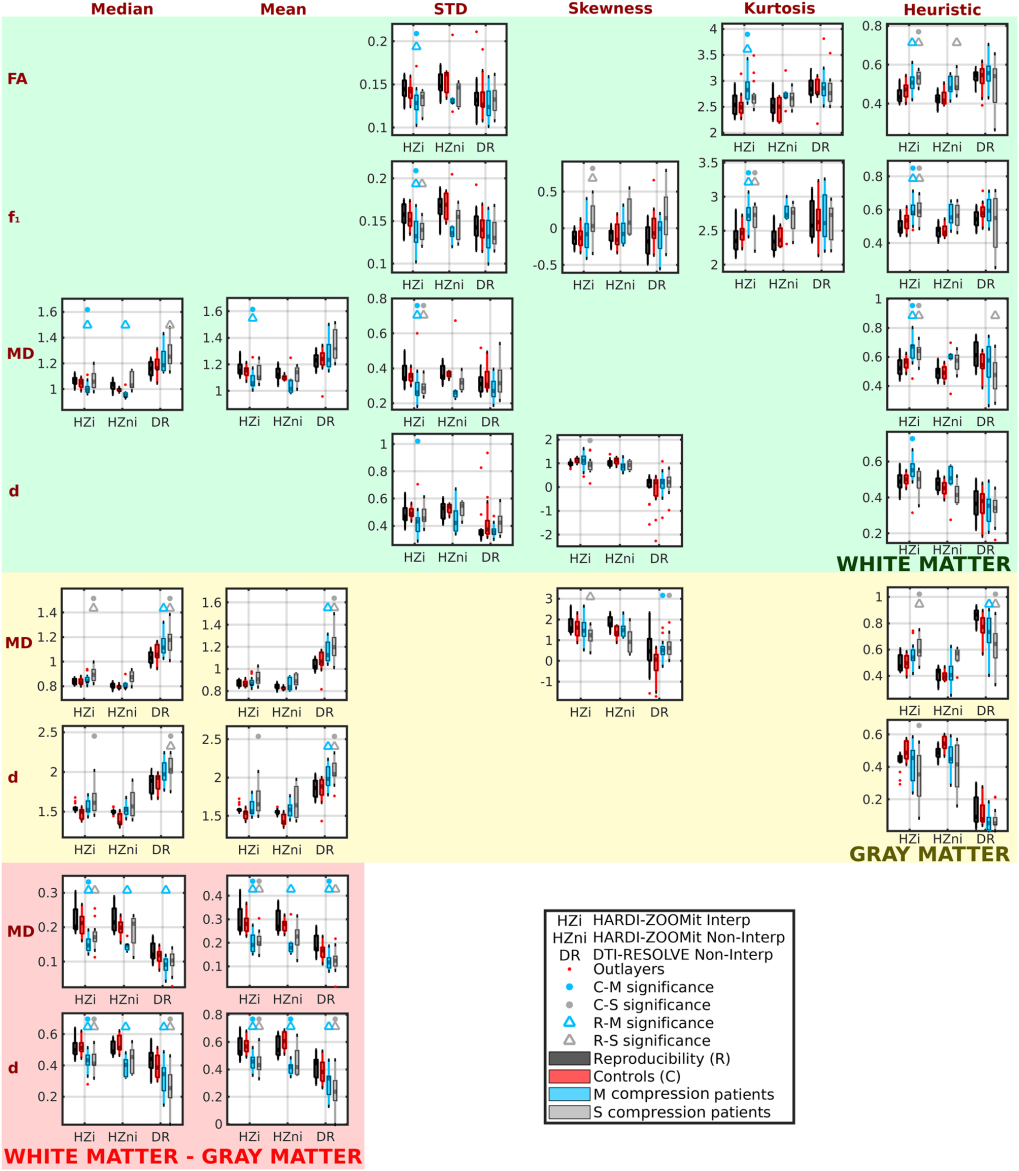
Diffusion MRI metrics with significant between-group differences ($p_{FWE}<$ 0.05) for age-comparable controls and mild (M) or severe (S) compression non-myelopathic patient groups. “Reproducibility” refers to a group of young healthy subjects measured twice during separate acquisition sessions. For clarity, graphs are not shown for the diffusion metrics for which control-patient between-group differences were not statistically significant.

For each subject and each dMRI metric, mean, median, standard deviation, skewness and kurtosis of the metric were evaluated within WM and GM, along with differences between means or medians in the WM and GM (e.g. $mean(MD)^{WM}-mean(MD)^{GM}$). Four different dMRI metrics (i.e. FA, $f_1$, MD and *d*) were analyzed and yielded 48 descriptive statistics parameters for each dMRI protocol. Findings for all parameters are presented in Supplementary Figs. S1, S2, S4, S5. Nineteen of the 48 parameters demonstrated significant differences ($p_{FWE}<$ 0.05) between age-comparable control (C) group and mild (M) or severe (S) compression non-myelopathic patients (Fig. 5; Table 1). Seventeen of the 19 parameters demonstrated significant differences for the HARDI-ZOOMit Interp protocol. The HARDI-ZOOMit Non-Interp protocol showed similar results (Fig. 5, Supplementary Figs. S1, S2, S4, S5), but with fewer significant findings likely due to the lower number of subjects scanned with this protocol. Eight of the 19 parameters were significantly different between controls and patients for the DTI-RESOLVE Non-Interp protocol (Fig. 5, Supplementary Figs. S1, S2, S4, S5). The DTI-RESOLVE only detected differences in the GM of NMDCCC patients and in differences between WM and GM values of the patients. Between-group differences were more robust in the HARDI-ZOOMit Interp protocol, when compared to the DTI-RESOLVE Non-Interp protocol, i.e. more significant observations with lower *p* values for the HARDI-ZOOMit (Fig. 5; Table 1, Supplementary Figs. S1, S2, S4, S5). We anticipated that the Ball and Stick and Stick model (providing local crossing models with relatively few parameters) might enhance the clinical benefit of the proposed HARDI-ZOOMit protocol. This assumption has been partly validated as the $f_1$ metric demonstrated lower *p* values (Wilcoxon rank-sum tests) than FA (Table 1). This is in line with previous observations of improved clinical correlation for generalized fractional anisotropy (GFA)^36^ estimated from Q-Ball imaging, in contrast to the DTI FA^7^.

Post-hoc ANCOVA test utilizing age as a confounding variable identified 14 of 17 variables to still demonstrate significant differences between age-comparable controls and NMDCCC patients for the HARDI-ZOOMit protocol (Table 2). Skewness of $f_1$ from WM, median of MD from WM and skewness of *d* from WM disappeared from the original list. For the DTI-RESOLVE protocol the number of variables increased from 8 to 9 (Table 2), again all are GM related or diffusivity gradient related as in the previous results. ANCOVA observations mostly overlap with the Wilcoxon rank-sum test observations (Fig. 5; Table 1).

**Table 2:** Post-hoc ANCOVA of dMRI metrics with significant differences between age-comparable healthy controls and NMDCCC patients.

Parameters	Shortcut	HARDI-ZOOMit Interp.	DTI-RESOLVE non-Interp.
		C vs M+S	C vs M+S
FA_WM_STD	FAwS	**8.43E−3**	3.27E−1
FA_WM_kurtosis	FAwK	**1.99E−3**	6.32E−1
f1_WM_STD	f1wS	**1.93E−3**	2.21E−1
f1_WM_skewness	f1wSK	7.84E−2	9.62E−1
f1_WM_kurtosis	f1wK	**9.46E−5**	8.46E−1
MD_WM_median	MDwM	6.64E−1	7.65E−2
MD_WM_mean	MDwm	**3.44E−2**	9.25E−2
MD_WM_STD	MDwS	**4.09E−3**	2.53E−1
d_WM_STD	dwS	**3.00E−2**	1.87E−1
d_WM_skewness	dwSK	8.28E−2	2.09E−1
MD_GM_median	MDgM	**3.25E−2**	**1.48E−2**
MD_GM_mean	MDgm	5.60E−2	**5.29E−3**
MD_GM_skewness	MDgSK	2.46E−1	**6.44E−4**
d_GM_median	dgM	**4.79E−3**	**3.66E−4**
d_GM_mean	dgm	**5.94E−3**	**1.49E−4**
MD_WM-GM_median	MDwgM	**2.07E−4**	**2.47E−2**
MD_WM-GM_mean	MDwgm	**3.48E−7**	**1.12E−3**
d_WM-GM_median	dwgM	**2.98E−5**	**2.26E−3**
d_WG-GM_mean	dwgm	**4.11E−7**	**1.64E−3**
FA_WM_heuristic	FAwH	**1.77E−3**	8.87E−1
f1_WM_heuristic	f1wH	**1.35E−3**	8.49E−1
MD_WM_heuristic	MDwH	**1.05E−3**	5.21E−1
d_WM_heuristic	dwH	3.27E−1	1.61E−1
MD_GM_heuristic	MDgH	**7.70E−3**	6.79E−2
d_GM_heuristic	dgH	**3.70E−3**	**2.24E−2**

**Figure 6:**
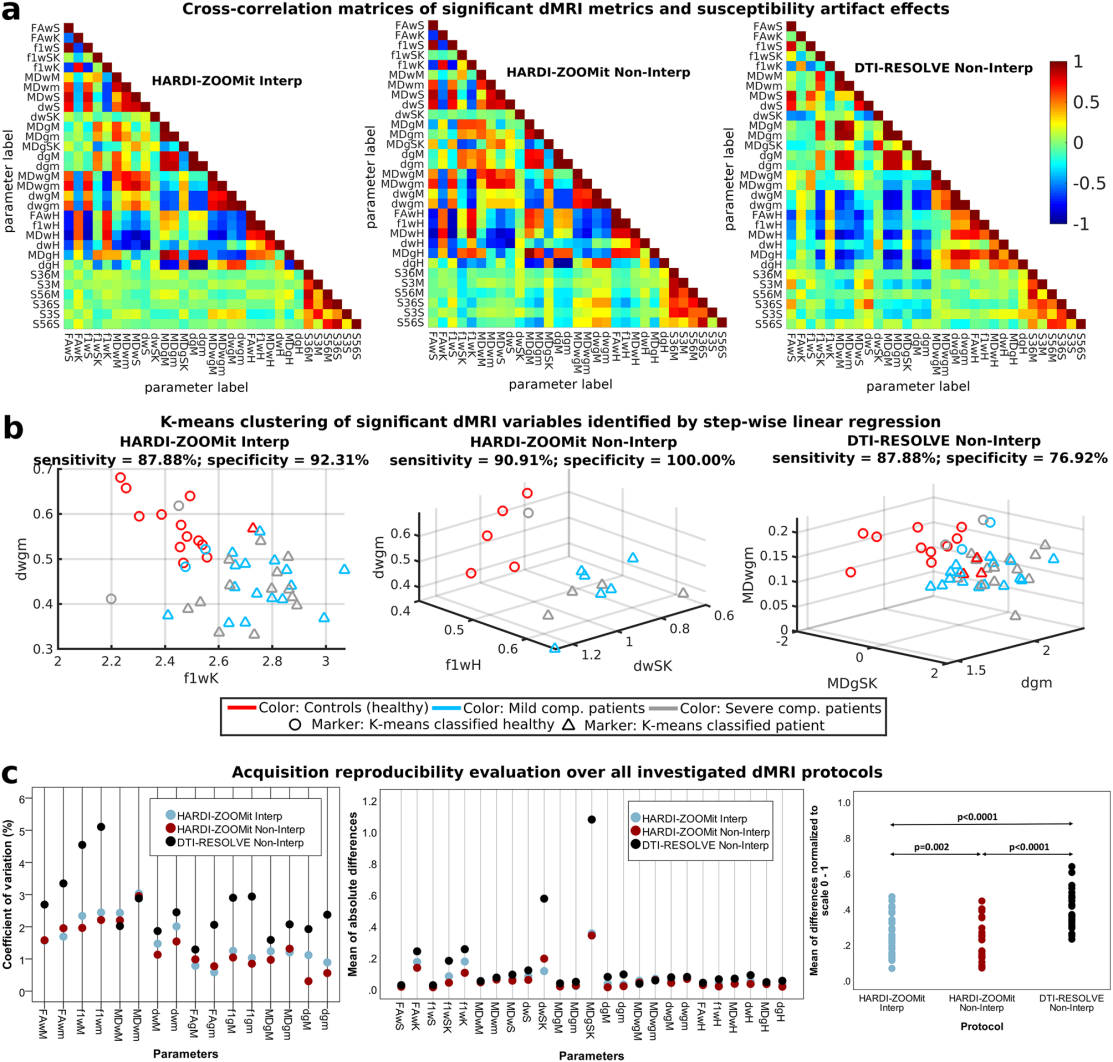
Cross-correlations of clinically significant diffusion MRI metrics and estimated susceptibility distortion parameters (**a**), K-means clustering of diffusion MRI metrics identified using step-wise linear regression (**b**) and reproducibility of diffusion MRI metrics over investigated protocols (**c**). Parameter names consists of 3 parts: (1) FA/f1/MD/d name of the diffusion MRI metric; (2) w/g/wg C3–C6 white matter, gray matter, white matter–gray matter difference respectively; (3) *M* median, *m* mean, *S* standard deviation, *SK* skewness, *K* kurtosis, *H* heuristic parameter. (**a**) The last six variables in matrices starting with letter S (e.g. S36M) represent measures of susceptibility artifact effects from three different ROIs (i.e. 36—C3–C6, 3—C3, 56—C5–C6).

List of parameters with their corresponding labels as used in Fig. 6 along with ANCOVA test *p* values for HARDI-ZOOMit Interp and DTI-RESOLVE Non-Interp protocols. Age was the confounding variable. Significant *p* values ($p<$ 0.05) are highlighted in bold. *C* age-comparable healthy controls, *M* mild NMDCCC patients, *S* severe NMDCCC patients.

For any investigated protocol or variable demonstrating significant differences between age-comparable healthy controls and mild or severe NMDCCC patients, post-hoc Wilcoxon rank-sum tests demonstrated no significant differences (i.e. each $\hbox {p}>0.05$) in descriptive statistics dMRI parameters of NMDCCC patients with or without radiculopathy.

### Novel heuristic parameters

Since the distributions in Fig. 3 demonstrated several dMRI metric intervals with disjunctive probability density functions (*g*), five heuristic parameters (*H*) of dMRI metrics (Eqs. 1–5) are proposed:

1 $$\begin{aligned} H_{FA}= & {} \int _{0.47}^{0.67} g(FA)dFA \end{aligned}$$

2 $$\begin{aligned} H_{f_1}= & {} \int _{0.30}^{0.55} g(f_1)df_1 \end{aligned}$$

3 $$\begin{aligned} H_{MD}= & {} \int _{0.84}^{1.26} g(MD)dMD \end{aligned}$$

4 $$\begin{aligned} H_{d_{WM}}= & {} \int _{1.70}^{2.20} g(d)dd \end{aligned}$$

5 $$\begin{aligned} H_{d_{GM}}= & {} \int _{1.00}^{1.48} g(d)dd \end{aligned}$$

All WM-based heuristic parameters (Eqs. 1–4) demonstrated significant clinical differences between control and patient groups (Fig. 5; Table 1) and $H_{MD}$ or $H_{dGM}$ in GM (Eqs. 3, 5; Fig. 5; Table 1) for the HARDI-ZOOMit Interp protocol. $H_{MD}$ measured in GM was significant for the DTI-RESOLVE Non-Interp protocol (Eq. 3; Fig. 5; Table 1). All estimated values of proposed heuristic parameters are shown in Supplementary Fig. S6. Post-hoc ANCOVA with age as a confounding variable rejected the $H_{dWM}$ measured in WM to demonstrate significant differences for the HARDI-ZOOMit protocol (Table 2). For the DTI-RESOLVE protocol, original $H_{MD}$ GM significance disappeared, but $H_{dGM}$ appeared significant (Table 2). Same as for the descriptive statistics parameters, the post-hoc Wilcoxon rank-sum tests demonstrated no significant differences in heuristic dMRI metrics of NMDCCC patients with or without radiculopathy.

### Step-wise linear regression and K-means clustering

Descriptive statistics and our heuristic approach identified 25 parameters with significant discrimination power between age-comparable controls and mild or severe NMDCCC patients with Wilcoxon rank-sum tests (Fig. 5; Table 1). Cross-subject cross-correlation matrices demonstrate high similarity between several pairs of dMRI metrics (Fig. 6a). Step-wise linear regression ($\mathbf{Y} =\beta _0+\mathbf{X} {} \mathbf{{\beta }}+\mathbf{{\epsilon }}$) identified a minimal linear mixture model maximizing separation between age-comparable healthy controls and all NMDCCC patients for each investigated dMRI protocol. Full visualization of the best model fits is shown in Supplementary Fig. S7 for each protocol. In descending order using Pearson correlation coefficient (*r*) between class signal (${\textbf{Y}}$) and predicted signal ($\mathbf{Y} _p=\mathbf{X} {} \mathbf{{\beta }}$):

HARDI-ZOOMit Non-Interp protocol demonstrated four significant variables, i.e. skewness value of *d* in WM (dwSK), white–gray matter gradient of *d* median values (dwgM), white–gray matter gradient of *d* mean values (dwgm) and heuristic parameter of $f_1$ in WM (f1wH). The model fit efficiency metrics were as follows: $r=0.892$, $RMSE=0.252$, model *F* value $F=10.74$, model *p* value $p=8.56\hbox {E}^{-4}$, model explained variance of $R^2=79.6\%$.

HARDI-ZOOMit Interp protocol demonstrated two significant variables (dwgm; and kurtosis of $f_1$ in WM—f1wK), with efficiency metrics: $r=0.733$, $RMSE=0.317$, $F=25.01$, $p=6.23\hbox {E}^{-8}$, $R^2=53.8\%$. There is an overlap with the HARDI-ZOOMit Non-Interp protocol, i.e. the dwgm variable.

DTI-RESOLVE Non-Interp protocol demonstrated three significant variables (skewness of MD in GM—MDgSK; mean value of *d* in GM—dgm; and white–gray matter gradient of MD mean values—MDwgm), with efficiency metrics: $r=0.670$, $RMSE=0.350$, $F=11.39$, $p=1.35\hbox {E}^{-5}$, $R^2=44.9\%$.

Although significant relationship between age and dMRI metrics was previously observed in a healthy aging population^37^ or in patients with degenerative cervical cord compression^16^, none of the models identified age variable as the model-significant variable in the present data, suggesting that the age-effect in the dataset is low.

Only regression-identified significant variables were used in the following K-means clustering using two classes (Fig. 6b). In sensitivity (SE) and specificity (SP) descending order, HARDI-ZOOMit Non-Interp protocol demonstrated SE = 90.91%, SP = 100.00%; HARDI-ZOOMit Interp protocol SE = 87.88%, SP = 92.31%; and DTI-RESOLVE Non-Interp protocol SE = 87.88%, SP = 76.92%. K-means approach wrongly classified some outlying NMDCCC patients as healthy controls (Fig. 6b). While the used K-means clustering algorithm estimates and finds a linear hyper-plane separating clusters in the data, more advanced algorithms estimating non-linear hyper-planes^38^ may likely improve the sensitivity for each protocol. We have not implemented such methods here, as they require additional datasets for training and the sample sizes of our datasets (46 or 16 data acquisitions) are rather limited to consider such approach. Using the full dataset for the classifier training (Fig. 6b) might also led to the classifier overfitting in all used protocols.

### Level of susceptibility artifacts in dMRI protocols

SC compression can increase local susceptibility off-resonance effects impacting the estimation of dMRI metrics. The level of the effects was evaluated in three different ROIs, i.e. C3–C6 (full FOV), C3 (area without compression) and C5–C6 (area with definite compression). These effects were not significantly different across groups in any ROI (Supplementary Fig. S8). HARDI-ZOOMit protocols were affected by off-resonance effects (group averages of single-subject median effects were $16\pm 8\,\hbox {Hz}$ at C3–C6, $14\pm 7\,\hbox {Hz}$ at C3, and $21\pm 13\,\hbox {Hz}$ at C5–C6 for Interp protocol, $5\pm 3\,\hbox {Hz}$ at C3–C6, $6\pm 4\,\hbox {Hz}$ at C3, and $6\pm 4\,\hbox {Hz}$ at C5–C6 for Non-Interp protocol) although to a lesser degree than the DTI-RESOLVE Non-Interp protocol ($20\pm 11\,\hbox {Hz}$ at C3–C6, $23\pm 16\,\hbox {Hz}$ at C3, and $22\pm 15\,\hbox {Hz}$ at C5–C6). Off-resonance effects of HARDI-ZOOMit Interp protocol were not correlated with dMRI derived parameters (Fig. 6a) with $|r|=0.09\pm 0.07$, $p_r\approx 0.49$, neither DTI-RESOLVE Non-Interp ($|r|=0.15\pm 0.11$, $p_r\approx 0.27$) and neither HARDI-ZOOMit Non-Interp ($|r|=0.16\pm 0.12$, $p_r\approx 0.48$). Still, the lowest cross-correlation effects are observed for the HARDI-ZOOMit Interp protocol (Fig. 6a).

### Reproducibility in young healthy subjects

Coefficients of variation (CV) for mean/median WM/GM dMRI metrics, and absolute or normalized mean differences in 25 significant dMRI metrics are shown in Fig. 6c. CV revealed a large difference between both HARDI-ZOOMit protocols and DTI-RESOLVE ($p=0.007$ for both), and no difference between HARDI-ZOOMit protocols, whereas the evaluation of normalized differences proved to be significant between all protocols ($p<0.0001$ between HARDI-ZOOMit vs. DTI-RESOLVE; $p=0.002$ between HARDI-ZOOMit protocols). The reproducibility of HARDI-ZOOMit protocols in comparison to the DTI-RESOLVE Non-Interp protocol was either comparable or higher as shown in Fig. 6c.

### HARDI-ZOOMit protocol and data analysis innovations

Our proposed HARDI-ZOOMit protocols yielded a larger amount of significant metrics with higher specificity when compared to the DTI-RESOLVE Non-Interp protocol (Figs. 3, 5, 6b; Tables 1, 2). The increased mutual information between microstructural maps and WM/GM structures (Fig. 2d) and better separation ability of primary and secondary fiber bundles (Fig. 4b) can be the improving key aspects.

In comparison to the previously used DTI-RESOLVE Non-Interp protocol, the proposed HARDI-ZOOMit protocol includes several modifications which possibly underlie the higher specificity of the protocol for the targeted clinical application. These include multi-shell gradient waveform sampling (enabling better separation of different microstructural compartments^31^) over spherical q-space, and higher maximal b-value ($1000\,\hbox {s mm}^{-2}$ vs $650\,\hbox {s mm}^{-2}$) increasing the diffusion weighting effects^3^ while retaining sufficient SNR (Fig. 1f). The fact that HARDI-ZOOMit protocols yielded higher group mean/median FA and $f_1$ values than the DTI-RESOLVE Non-Interp protocol (Fig. 3, Supplementary Figs. S1, S2) supports the hypothesis that the data are less noisy, consistent with the fact that the cervical SC largely consists of fiber bundles oriented in the superior–inferior direction with high level of diffusion anisotropy expected.

We proposed the use of skewness, kurtosis and heuristic parameters extracted from subject’s cervical dMRI maps as robust statistic parameters in SC injury revealing significant differences between controls and patients (Figs. 5, 6b; Tables 1, 2). Also, while using the subtraction between WM and GM mean/median dMRI metrics, we have demonstrated the most significant inter-group differences (Figs. 5, 6b; Tables 1, 2). The subtraction method is comparable to a simple gradient approximation of dMRI metrics between WM and GM, and is a novel clinically feasible over all tested protocols for MD and *d* metrics.

Since we used the contiguous C3–C6 ROI space (i.e. data at all spinal levels), the obtained results have higher statistical power than previous studies in NMDCCC typically analyzing dMRI from several separate slices, mostly focused on the maximum compression level and compared to non-compressed levels^20,35^. Our approach addresses the issue of different ROI selection across subjects determined by the presence of SC compression at different segments but also the issue of physiologic dMRI metrics variation in different SC segments^39^. Moreover, the current approach minimizes artifact effects (Fig. 6a) potentially emphasized (Fig. 1f) in areas with the most severe compression.

### Limitations and future work

Although C3–C6 WM or GM ROIs are quite large and coarse, it is feasible to proceed to finer analysis of tract-specific dMRI metrics utilizing a template and tract-specific atlas co-registration^37,40^. Tract-specific changes within our studied group of patients might be investigated, as demonstrated in the case of DCM^41–43^ or ALS^44^ patients.

A HARDI-RESOLVE dMRI protocol with exactly the same gradient waveform samplings, same b-values, and same spatial resolution should be used for the optimal protocol comparison. Such protocol might increase the percentage of observed crossings (Fig. 4b), increase FA and $f_1$ group medians (Supplementary Figs. S1, S2), and even increase the protocol’s sensitivity or specificity to the presymptomatic myelopathy detection. However, the acquisition time of HARDI-RESOLVE protocol would significantly increase in comparison to the original 16 min for the DTI-RESOLVE protocol or 12.5 min for the introduced HARDI-ZOOMit protocols.

Further HARDI-ZOOMit protocol optimization or further increase in the protocol’s angular resolution may yield dMRI protocol visualizing SC crossing fibers in-vivo in clinically applicable acquisition time.

Diffusion weighted MR signal loss in areas close to the lungs (i.e. lower part of C7 and T1 acquired here; Fig. 1c,e) crucially limits the usability of the proposed HARDI-ZOOMit protocol for other regions apart from the cervical spine and SC. Suppression of the lung-induced artifacts will be the topic of future research that might extend the usability of the protocol to broader clinical applications imaging beyond cervical SC segments.

We showed that the proposed protocol was able to distinguish age-comparable healthy controls and presymptomatic patients with microstructural myelopathy. Changes in dMRI parameters for DCM patients with advanced clinical symptoms have been already reported^12–18^. Extending the dataset with groups of symptomatic DCM patients spanning from mild to severe cervical SC compressions to delineate the disease progression in all DCM stages will be addressed in upcoming research. Also, step-wise multiple linear regression was used in a relatively simple context, dividing four different groups into two classes (controls or patients). More advanced classification, e.g. patients with mild or severe SC compressions, has not been applied, and should be carried out in the future, along with exploration of the 25 parameters that rendered significant between-group differences (Fig. 5; Table 1). Our results might be influenced by the gender-related difference in the control group (i.e. 9 females and 4 males). However, since the control group results overlapped with the young healthy participant group (i.e. reproducibility group, Fig. 3), we have assumed that gender effect was minimal. Similarly as we assumed it for the age-effect, which we have also confirmed with the ANCOVA test (Table 2). In contrast to the DTI-RESOLVE protocol, the presented HARDI-ZOOMit protocol has not demonstrated so many significant changes for MD or *d* in the GM (Fig. 5; Tables 1, 2). If these findings are confirmed, the clinical DTI-RESOLVE protocol provides additional information about the GM microstructural changes.

## Discussion

Using an optimized HARDI-ZOOMit dMRI protocol and a “*high SNR*” DTI-RESOLVE protocol^33^, we demonstrated a global difference of diffusion MRI metrics over contiguous C3–C6 levels between healthy controls and NMDCCC patients underlying presymptomatic microstructural myelopathy. The *practical impact* of detecting subclinical microstructural SC injury in NMDCCC using fast, reliable and clinically feasible dMRI protocol is significant. First, in addition to other quantitative MRI methods (i.e. magnetization transfer imaging and $\hbox {T}_2^*$-weighted imaging), it verifies and quantifies microstructural SC involvement in subjects with compressed spinal cord due to degenerative cervical stenosis^19^. Such knowledge opens the door for more accurate definition of the myelopathy diagnosis which is currently equivocal and often based on clinical symptoms, which appear relatively late. DCM is already symptomatic and clinical deficits may often be irreversible. Second, the time course and reversibility of microstructural dMRI-detected SC changes in the compressed SC and their correlation with similar “*functional*” and possibly “*biochemical*” changes detected in NMDCCC using evoked potentials and magnetic resonance spectroscopycite^45,46^ may further describe the DCM pathophysiology and lead to the optimization of the DCM diagnosis. Finally, these changes may help to identify NMDCCC subjects with higher risk of developing DCM (i.e., “*presymptomatic myelopathy*”), in addition to already demonstrated predictors^35,45^.

The DTI-RESOLVE Non-Interp scans were occasionally contaminated by insufficient fat suppression or aliasing artifacts (e.g. in the upper parts of the FOV with voxels originating from areas outside the FOV, from subjects’ chin or adipose tissue in particular). While the artifacts were mostly present on the outside of the SC ROI, their effect on the dMRI metrics should be minimal. It is also important to note that the performance of HARDI-ZOOMit protocol remains limited for the SC imaging at and below C7 vertebra.

Major fiber tracts in the cervical SC are highly organized along the SC longitudinal axis resulting in relatively high FA or $f_1$ values. Our finding of higher FA and $f_1$ mean/median value estimates with HARDI-ZOOMit protocols suggests that the HARDI-ZOOMit data are less noisy than DTI-RESOLVE protocol data. Higher statistical power of between-group differences for $f_1$ derived statistical parameters (compared to complementary FA parameters) demonstrated the efficiency of the multi-shell data acquisition and additive multi-compartment microstructural models (estimating local fiber crossings) and the potential applicability in the clinical quantitative diagnostic imaging. Further clinical HARDI protocol optimization utilizing other shells with $\hbox {b}>1000\,\hbox {s mm}^{-2}$, sufficient SNR and sufficient acquisition time would permit using other advanced microstructural models such as NODDI (neurite orientation dispersion and density imaging)^47–50^, MIX (microstructure imaging of crossing)^51^, CHARMED (composite hindered and restricted model of diffusion)^52^, AxCaliber^53^, ActiveAx^54^ or direct multi-shell multi-tissue constrained spherical deconvolution^55^, etc. Default implementations of such methods expect high b-values or even variable diffusion $\delta /\Delta$ times within the same shell. Although low b-value single-shell SC implementation exists for NODDI^56^, it remained beyond the focus of our already quite extensive analysis. The current data sets and pipeline codes were made publicly available, so, researchers may investigate and compare other diffusion models.

## Methods

### Participants

All participants signed an informed consent enabling the experimentation with human subjects and were enrolled in the study that was approved by the *Masaryk University* (*Brno, Czech Republic*) ethics committee and by the *University Hospital Brno* (*Brno, Czech Republic*) ethics committee, both in concordance with the Declaration of Helsinki. NMDCCC patients were identified in an extensive sample of the Caucasian population of the South Moravia region, through an epidemiological study assessing the prevalence of degenerative cervical cord compression^21^. All participants underwent MRI examination of the cervical spine on a 1.5 T MR Philips Achieva scanner with a standard 16-channel head and neck coil. The standardized imaging protocol included conventional pulse sequences in sagittal-$\hbox {T}_1$, $\hbox {T}_2$ and short-tau inversion recovery (STIR), and axial planes (gradient-echo $\hbox {T}_2$) for the purpose of morphological evaluation. The clinical status of patients/healthy controls was blinded to two neuroradiologists who evaluated and agreed on the assessment of the compression in the majority of cases. Where disagreement existed—seldom—the final decision was based on a consensus. The imaging criterion for cervical cord compression, dividing groups at healthy controls and NMDCCC patients, was defined as a change in SC contour or shape at the level of an intervertebral disc on axial or sagittal MRI scan compared to that at midpoint level of neighboring vertebrae. NMDCCC patients were then clinically examined by two certified neurologists (experienced in degenerative cervical myelopathy diagnosis) who excluded the presence of any clinical myelopathic signs or symptoms^35^.

In addition to the assessment of cervical cord compression, the following conventional MRI parameters were also measured to quantify the severity of compression: cross-sectional area (CSA) of the SC and compression ratio (CR) calculated as anteroposterior/laterolateral SC diameter. Severe SC compressions were defined as both $\hbox {CSA}\le 70\,\hbox {mm}^2$ and $\hbox {CR}\le 0.4$. These thresholds proved to increase the risk of development of symptomatic DCM in a previous study^35^.

Several young healthy volunteers were investigated twice with an inter-scan interval (> 1 day) to evaluate test–retest reliability of used imaging methods, i.e., reproducibility. Young healthy volunteers passed neither radiological nor neurological evaluation. The inter-scan interval ranged from 0 to 29 weeks (mean ± standard deviation $7\pm 11$ weeks).

### MRI acquisition

High-resolution sagittal $\hbox {T}_2$-weighted ($\hbox {T}_2\hbox {w}$) and axial $\hbox {T}_2^*$-weighted ($\hbox {T}_2^*\hbox {w}$) anatomical scans were acquired followed by two independent axial dMRI acquisition protocols (i.e. HARDI-ZOOMit Interp and DTI-RESOLVE Non-Interp) in randomized order to disperse the effect of motion artifacts uniformly over sessions. In a subset of study participants, the HARDI-ZOOMit Non-Interp protocol was also acquired. All imaging was performed on a 3T scanner (Magnetom Prisma; *Siemens Healthcare, Erlangen, Germany*) using the standard 64-channel head/neck and the 32-channel spine coils. All axial sequences were pulse triggered increasing the expected data acquisition time (TA) about 10–15% for each sequence.

$\hbox {T}_2$*-weighted sagittal images* were acquired to cover the whole cervical SC with 30 contiguous slices using a turbo spin-echo sequence with $\hbox {TR}=8640\,\hbox {ms}$ (repetition time), $\hbox {TE}=98\,\hbox {ms}$ (echo time), 4 averages, $\hbox {GRAPPA}=2$, field of view (FOV) $250\times 250\,\hbox {mm}^2$, matrix size $896\times 896$ voxels, slice thickness 1.3mm, voxel size $0.28\times 1.30\times 0.28\,\hbox {mm}^3$. The acquisition time (TA) was 8min 49s.

$\hbox {T}_2^*$*-weighted axial images* were acquired to cover the C3–C7 levels with 42 contiguous slices using a MEDIC (multi-echo data image combination) sequence with $\hbox {TR}=778\,\hbox {ms}$, $\hbox {TE}=17\,\hbox {ms}$ (4 echoes), 2 averages, FOV $180\times 180\,\hbox {mm}^2$, matrix size $512\times 512$ voxels after interpolation in Fourier domain, slice thickness 2.5 mm, voxel size $0.70\times 0.70\times 2.50\,\hbox {mm}^3$ (re-sampled to $0.35\times 0.35\times 2.50\,\hbox {mm}^3$ within MR scanner image reconstruction), TA would be 7 min 51 s, if pulse trigger was not used.

*HARDI-ZOOMit interpolated dMRI protocol* was acquired to cover the C3–C7 levels with 35 contiguous axial slices with $\hbox {TR}=6700\,\hbox {ms}$, $\hbox {TE}=73\,\hbox {ms}$, $\hbox {FOV}=44\times 129\,\hbox {mm}^2$, matrix size $68\times 200$ voxels, slice thickness 3mm, voxel size $1.30\times 1.30\times 3.00\,\hbox {mm}^3$ re-sampled to $0.65\times 0.65\times 3.00\,\hbox {mm}^3$ after interpolation in Fourier domain. Sixty-three diffusion weighted images (42 gradient directions with $\hbox {b-value} = 1000\,\hbox {s mm}^{-2}$ and 21 directions with $\hbox {b}=550\,\hbox {s mm}^{-2}$) and 7 images ($\hbox {b}_0$) with $\hbox {b}=0\,\hbox {s mm}^{-2}$ were collected with anterior-posterior (AP) phase encoding. Five additional $\hbox {b}_0$ images were acquired using posterior–anterior (PA) phase encoding. A total expected TA without pulse trigger would be 12 min 46 s. The 63 gradient directions were uniformly sampled over two spherical shells (see Fig. 4a) with Caruyer et al.’s^57^ sampler.

*HARDI-ZOOMit non-interpolated dMRI protocol* was acquired with the same protocol without the interpolation in Fourier domain and with the same acquisition time. The matrix size decreased at $34\times 100$ voxels and voxel size stayed $1.30\times 1.30\times 3.00\,\hbox {mm}^3$.

*DTI-RESOLVE dMRI protocol* consisted of two identical sessions with opposite phase encodings (AP, PA). For each encoding, 30 diffusion weighted images with $\hbox {b}=650\,\hbox {s mm}^{-2}$ and $5\,\hbox {b}_0$ images were collected. The acquired data cover C3–C7 levels with 30 contiguous axial slices with $\hbox {TR}=4500\,\hbox {ms}$, $\hbox {TE}_1=50\,\hbox {ms}$, $\hbox {TE}_2=77\,\hbox {ms}$, $\hbox {FOV}=73\times 165\,\hbox {mm}^2$, matrix size $66\times 118$ voxels, slice thickness 3.30 mm, voxel size $1.10\times 1.10\times 3.30\,\hbox {mm}^3$. TA without pulse trigger would be 16 min 16 s.

All MRI acquisition protocol files (i.e. .pdf file with MRI parameters, .exar1 file for an easy upload into the Siemens MR console and .dvs file with HARDI sampling) are stored under the URL link listed in the *Data availability* section.

### dMRI protocol’s SNR estimation

For each data acquisition and each dMRI protocol separately, DWI and $\hbox {b}_0$ scans were separated and extracted. *Without any data preprocessing*, mean DWI and mean $\hbox {b}_0$ images were estimated. SC was segmented with sct_deepseg_sc^58^ algorithm from the mean DWI image. If the protocol consisted of more than one *q-space* shells, DWI data were separated at each single-shell. The first four and last four slices were excluded from estimation of the mean intensity ($I_{SC}$) of DWI signal inside SC of each shell. Air area was located in mean $\hbox {b}_0$ image of each participant with thresholding of the superior half of the FOV. Noise standard deviation ($\sigma _{air}$) was estimated from segmented air area. The SNR was estimated with Eq. (6) optimized or estimation from two different ROIs^59^. SNR group medians, means and STDs evaluated the level of the noise of each shell and each protocol.

6 $$\begin{aligned} SNR = \frac{I_{SC}}{\sqrt{\frac{2}{4-\pi }}\sigma _{air}} \end{aligned}$$

### Data processing pipeline

The acquired data were processed with Spinal Cord Toolbox 3.2.3 (SCT)^60^, ANTs 2.1.0 (Advanced Normalization Tools)^61^ and and FSL 5.0.10 (FMRIB Software Library)^62^ software libraries implemented all together within in-house made bash scripts that also include some in-house routines programmed in MATLAB (*MathWorks, Natick, USA*).

Areas outside the body were removed from sagittal and axial anatomical scans with low intensity thresholding and both scans were bias-field corrected^63^. Since the axial $\hbox {T}_2^*$ data were acquired with inter-leaved data collection process, the data were corrected slice-by-slice with in-house implemented algorithm utilizing affine registeration^64^ and additive fusion^64^ of even and odd slices. Sagittal scan was re-sampled at voxel size $0.28\times 0.35\times 0.28\,\hbox {mm}^3$ resolution and cropped to cover only a cervical area (i.e. T2SAG space). SC was initially segmented from T2SAG scan with a sct_deepseg_sc^58^ algorithm implemented in SCT^60^, z-axis slice number containing C2/C3 disc was manually marked, and sct_label_vertebrae^65^ library automatically labeled individual vertebral levels. The bias-field corrected T2SAG image was co-registered with fixed axial image (i.e. T2TRA space), using sct_register_multimodal^60^ script utilizing series of ANTs^61^ registration algorithms (initially optimized for brain image registrations)^66^. Segmented SC and its labels were warped from sagittal T2SAG space into co-registered T2TRA space. The 2^nd^ iteration of SC segmentation^58^ and labelling^65^ was performed in the T2TRA space, following with GM segmentation utilized with sct_deepseg_gm^67^ library. WM area was obtained by subtraction of the SC and GM masks. All segmented masks and vertebral labelings in T2TRA space were then visually inspected and corrected if necessary.

dMRI data of each protocol were processed separately, utilizing the same pipeline, as follows: Susceptibility, motion and eddy currents artifacts were minimized from the entire FOV with FSL^62^ topup^68^ and eddy^69^ functions. From the preprocessed dMRI data, DTI^3^ and ball and stick^30^ models were estimated with dtifit and bedpostx functions, both implemented in FSL^62^. From DTI estimates, fractional anisotropy (FA)^3^ and mean diffusivity (MD)^3^ maps were derived for each subject. For the ball and stick models, two crossing fiber bundles with their partial volume fractions ($f_1,f_2$)^30^ were expected as a maximal occurring number (i.e. Ball and Stick and Stick model), and single mean diffusivity values (*d*)^30^ without any variance were estimated per each voxel. Single-subject FA and $f_1$ maps are complementary, similarly to MD and *d* maps. From eddy output (4D image), DWIs, $\hbox {b}_0$ images and their mean versions were extracted and separated by sct_dmri_separate_b0_and_dwi^60^ function. SC in diffusion space was segmented from the “DWI_mean” image again with the sct_deepseg_sc^58^ function. Utilizing the sct_register_multimodal^60^ command, a single-subject “$\hbox {b}_0$_mean” image was co-registered into T2TRA space with fixed $\hbox {T}_2^*$-w axial image, while segmented masks of SC from DWI_mean and bias-field corrected $\hbox {T}_2^*$-w axial images were used to define the regions of interest (ROIs). Estimated warping field was then used for geometrical transformations of all dMRI metrics (i.e. FA, MD, $f_1$, *d*, etc.) from diffusion space of each protocol into a T2TRA space. In this space, two different ROIs were defined (using segmented masks and labels), i.e. C3–C6 WM area and C3–C6 GM area. SC at level C3-6 was analyzed as C3–C6 area is the most often affected by SC compression^35^. Diffusion MRI-derived quantitative parameters from the C3–C6 area were compared over 4 different groups of subjects and over three different acquired dMRI protocols, as described in following section.

### Mutual information between dMRI metrics and WM/GM structures

Mutual information is a similarity criterion detecting increased magnitude for similar images with both linear or non-linear contrast transform functions^64^. Considering a non-linear transform function between microstructural maps and WM/GM structures, non-normalized mutual information (*I*, Eq. 7)^64^ was estimated between each dMRI metric map (*a*, i.e. FA, $f_1$, MD or *d*) and semi-binarized $\hbox {T}_2^*$w axial image (*b*, background = 0; GM = 1; and WM = 2) inside C3–C6 SC area for each subject and protocol. Variable $E_a$ represent the entropy of image *a*, $E_b$ entropy of image *b*, and $E_{ab}$ the joint entropy between images *a* and *b*^64^. Function *s*(*a*) is the histogram of image *a* with intensity indexes from 1 to *q*. Function *u*(*b*) is the histogram of image *b* with intensity indexes from 1 to *r*. Function *v*(*a*, *b*) is the joint histogram^64^ between images *a* and *b*. Similarity criterion distributions were visualized for each dMRI metric and protocol, and values of distributions’ group-averaged quartiles and medians were used for the description and evaluation of the rate of mutual entropy^64^ between dMRI metric maps and WM/GM structures over protocols.

7 $$\begin{aligned} I = E_a + E_b - E_{ab} = -\sum _{l=1}^q s(a_l) \log ( s(a_l) ) -\sum _{m=1}^r u(b_m) \log ( u(b_m) ) + \sum _{l=1}^q \sum _{m=1}^r v(a_l,b_m) \log ( v(a_l,b_m) ) \end{aligned}$$

### Quantitative measurements from ROIs, group-level and inter-protocol comparisons

ROIs were characterized with several parameters of descriptive statistics usually estimated for random variables (i.e. estimated dMRI metrics: FA, MD, $f_1$ and *d* maps). They were the first four moments (i.e. mean, standard deviation - STD, skewness and kurtosis) of Gaussian probability density function (p.d.f.) and median. For each dMRI protocol separately, Wilcoxon rank-sum test was used to investigate whether a group median of some dMRI parameter differed, especially between some group of patients and group of age-comparable controls. Critical *p* value was set to $p<0.00833 \approx p_{FWE}<0.05$, since there are six possible different comparisons over four groups of subjects for one dMRI protocol and $0.05/6=8.33\hbox {E}^{-3}$ (FWE—family wise error correction for multiple comparisons).

Because the Gaussian approximation often does not provide a good fit to the measured data especially for random variables with non-symmetric p.d.f.s, we have fitted smooth p.d.f.s directly from a histogram of each ROI with a “normal” kernel (as implemented in MATLAB with fitdist function). Mean p.d.f.s with Q1–Q3 confidence intervals (Q—quartile) were derived for each group of subjects and each protocol. Heuristic parameters (*H*) which may clinically differentiate controls and non-myelopathic patients were proposed as Eq. (8). Values $x_1$ and $x_2$ are marginal values of a dMRI metric (*x*), where confidence intervals are disjunctive for derived smooth p.d.f.s (*g*).

8 $$\begin{aligned} H_{x} = \int _{x_1}^{x_2} g(x)dx \end{aligned}$$

Wilcoxon rank-sum test was used for testing differences between groups of subjects in the same way as for descriptive statistics parameters.

To minimize age-effect in the comparison results, post-hoc ANCOVA (analysis of covariance) was used as an additional between-group difference test where age was used as a confounding variable. One tested group were age-comparable healthy controls, second group were all NMDCCC patients. If test’s $p<0.05$, the between-group difference was considered to be significant.

To evaluate the effect of radiculopathy, post-hoc Wilcoxon rank-sum tests investigated the differences in statistical or heuristic dMRI metrics of NMDCCC patients with or without radiculopathy. Significance level was set at $p<0.05$.

### Diffusion MRI metric redundancy, uniqueness, sensitivity and specificity

Cross-subject Pearson correlation coefficients (*r*) evaluated level of linear dependence between dMRI metrics demonstrating significant between-group differences. Step-wise linear regression (${\textbf{Y}}=\beta _0+\varvec{X\beta }+\varvec{\epsilon }$) was used to identify unique dMRI metrics that maximize differences between age-comparable healthy control (C) and NMDCCC patient (M, i.e. mild compression and S, i.e. severe compression) groups. ${\textbf{Y}}$ is a vector equal to 0.5 for positions of C subjects and −0.5 to positions of M or S subjects. Significant variables (i.e. dMRI metrics) in model matrix ${\textbf{X}}$ were added based on their variable *p* values ($<0.05$) quantifying belonging to the final model fit. Because of an age effect concerns, we have also added the age variable as the tested parameter (i.e. potential significant compartment of the final matrix ${\textbf{X}}$). Set of significant variables in model matrix ${\textbf{X}}$ was used as an input feature into automatic K-means clustering at 2 classes. Sensitivity (SE) and specificity (SP) of each dMRI protocol was evaluated by comparison of the K-means classification with control-patient classification done by radiologist and neurologist experts.

### Level of off-resonance effects in dMRI data

Single-subject mean, median, and STD of absolute off-resonance effects (i.e. field coefficient output of the topup^68^ function) were estimated for each data acquisition from three different SC ROIs (C3–C6 characterizing our dMRI analysis ROI, C3 characterizing area without probable compression, and C5–C6 characterizing area with possible compression in patients) defined by segmented and labeled SC of $\hbox {T}_2^*\hbox {w}$ axial scan. Differences over subject groups were tested again using Wilcoxon rank-sum test. Cross-subject Pearson correlation coefficients (*r*) evaluated cross-correlations with dMRI derived parameters observing significant differences between control and patient groups to test whether the observed difference is/is not caused by different level of off-resonance effects.

### Test–retest reliability of dMRI protocols

Test–retest reliability (i.e. reproducibility) was tested in a group of seven young healthy volunteers who were scanned twice with time interval between session ranging from 0 to 29 weeks (mean ± standard deviation 7 ± 11 weeks). The minimum distance was 1 day. Mean coefficients of variation (CV, the ratio of standard deviation to the mean of repeated measures) were calculated for parameters expressing single-subject WM/GM dMRI metric mean or median. Absolute differences between consecutive measurements in seven subjects were calculated for all dMRI parameters where significant differences between controls and a patient group were observed. For a comprehensive comparison of protocols, a min–max normalization to range 0–1 was employed on each variable difference through all three protocols. Means of normalized differences were calculated for each variable per protocol. Three different protocols were then compared by CVs and means of normalized differences using series of Wilcoxon signed ranks tests with Bonferroni correction for multiple testing. Statistical testing was performed in SPSS version 23 (*IBM, Armonk, New York*).

## Supplementary information

Supplementary material 1

## Data Availability

Acquired MRI data reported in the manuscript, a table with basic participants’ demographics, and “*HARDI-ZOOMit.pdf*” and “*HARDI-ZOOMit.exar1*” files with used MRI protocol parameters are available at the URL: https://hdl.handle.net/20.500.12618/0000-5c13d342-4798-41d9-8d2a-bf750ab79fdb.

## Code availability

All in-house implemented codes and scripts with the installation manual are available at the URL: https://github.com/renelabounek/sc-dmri-myelopathy.
