# Supplementary material for: HARDI-ZOOMit protocol improves specificity to microstructural changes in presymptomatic myelopathy
Source: Sci Rep. 2020 Oct 16;10:17529. doi: 10.1038/s41598-020-70297-3 (PMC7567840; doi:10.1038/s41598-020-70297-3)
Supplement: Supplementary file 1 — Supplementary material 1 [file 41598_2020_70297_MOESM1_ESM.pdf]

Supplementary Material

HARDI-ZOOMit protocol improves specificity to microstructural changes in presymptomatic myelopathy

René Labounek[1,2,3], Jan Valošek[1,2], Tomáš Horák[4,5], Alena Svátková[4,6], Petr Bednařík[4,7], Lubomír Vojtíšek[4], Magda Horáková[4,5], Igor Nestrašil[3,8], Christophe Lenglet[8], Julien Cohen-Adad[9], Josef Bednařík[4,5], Petr Hluštík[2,10,*]

[1] *Department of Biomedical Engineering, University Hospital Olomouc, Olomouc, Czech Republic*
[2] *Department of Neurology, Palacký University, Olomouc, Czech Republic*
[3] *Division of Clinical Behavioral Neuroscience, Department of Pediatrics, University of Minnesota, Minneapolis, MN, USA*
[4] *Central European Institute of Technology, Masaryk University, Brno, Czech Republic*
[5] *Department of Neurology, University Hospital Brno, Czech Republic*
[6] *Department of Medicine III, Clinical Division of Endocrinology and Metabolism, Medical University of Vienna, Vienna, Austria*
[7] *High Field MR Centre, Medical University of Vienna, Vienna, Austria*
[8] *Center for Magnetic Resonance Research, Department of Radiology, University of Minnesota, Minneapolis, MN, USA*
[9] *Institute of Biomedical Engineering, Polytechnique Montreal, Montreal, Canada*
[10] *Department of Neurology, University Hospital Olomouc, Olomouc, Czech Republic*

***Corresponding authors:** Petr Hluštík, phlustik@upol.cz

Document description

The document consist of eight supplementary figures visualizing results in more detail. The manuscript refers the supplementary figures in the text.

FigS. 1 displays graphs of all single-acquisition results with all descriptive statistics parameters (i.e. median, mean, standard deviation, skewness, and kurtosis) derived from white / gray matter (WM/GM) fractional anisotropy (FA) maps, and graphs of WM-GM subtraction (i.e. gradient between WM and GM) for FA means and medians. All graphs include p-values of all Wilcoxon rank-sum tests only when the between-group test was considered significant (i.e. $p_{FWE}<0.05\approx8.33e^{-3}$).

FigS. 2 displays partial volume of the 1st anisotropic diffusion principal direction (f_1) maps. The same result visualization/description was used as in **FigS. 1** for FA maps.

FigS. 3 displays the smooth p.d.f.s of investigated dMRI metrics from C3-C6 gray matter.

FigS. 4 displays mean diffusivity (MD) maps of DTI model. The same result visualization/description was used as in **FigS. 1** for FA maps.

FigS. 5 displays mean diffusivity maps (d) of Ball & Stick & Stick model. The same result visualization/description was used as in **FigS. 1** for FA maps.

FigS. 6 displays for heuristic parameters. The same result visualization/description was used as in **FigS. 1** for FA maps.

FigS. 7 displays the full result visualization of step-wise linear regression of dMRI metric parameters observing significant difference between healthy controls and group of mild or severe NMDCCC patients across all investigated dMRI protocols.

FigS. 8 displays evaluations of susceptibility to off-resonance effects across all investigated dMRI protocols.

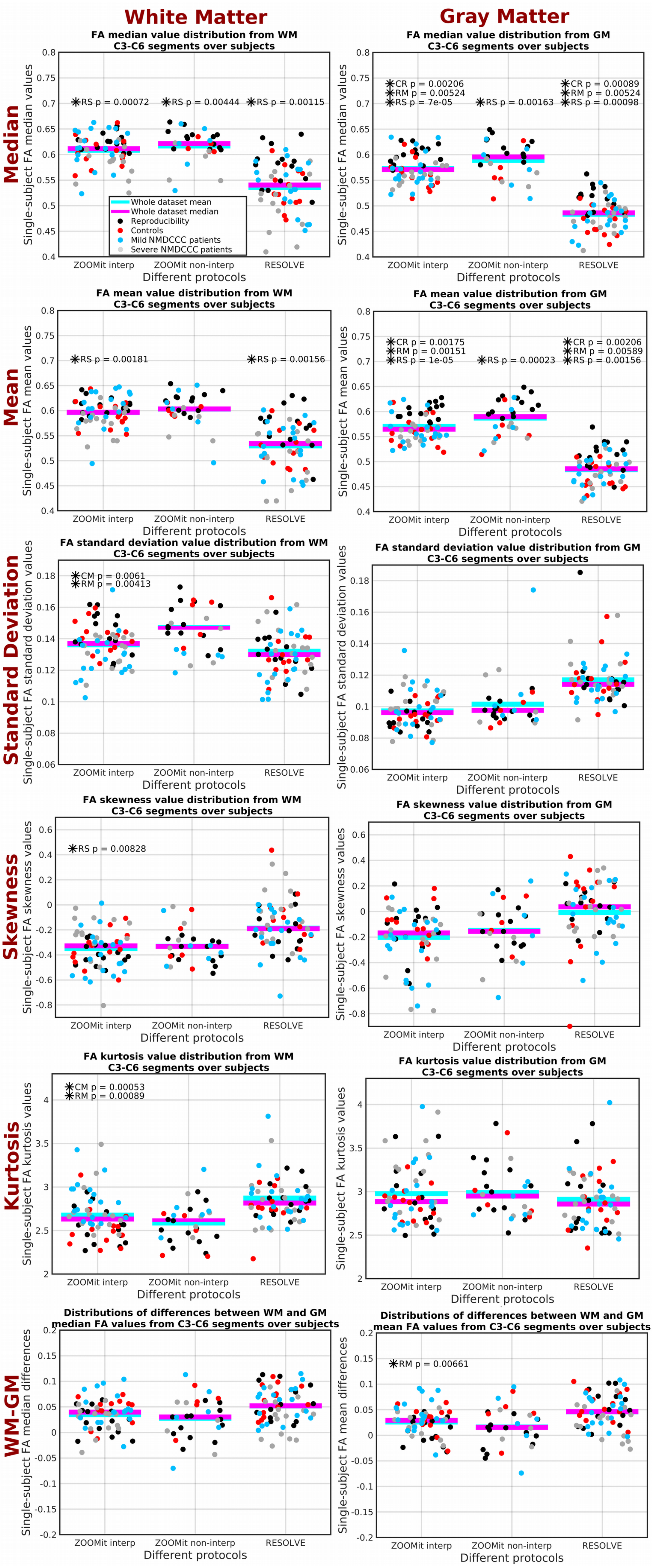

FigS. 1 | Descriptive statistics and WM/GM mean/median gradient results for FA maps of three investigated dMRI protocols. Each dot represents a result of a single data acquisition. Study groups are color-coded see caption. P-values are only displayed when the Wilcoxon rank-sum test indicated a significant between-group difference. Group denotation: C - age-comparable control group, R - young healthy volunteers measured twice for protocol reproducibility evaluations, M - patients with mild non-myelopathic degenerative cervical cord compression (NMDCCC), S - patients with severe NMDCCC.

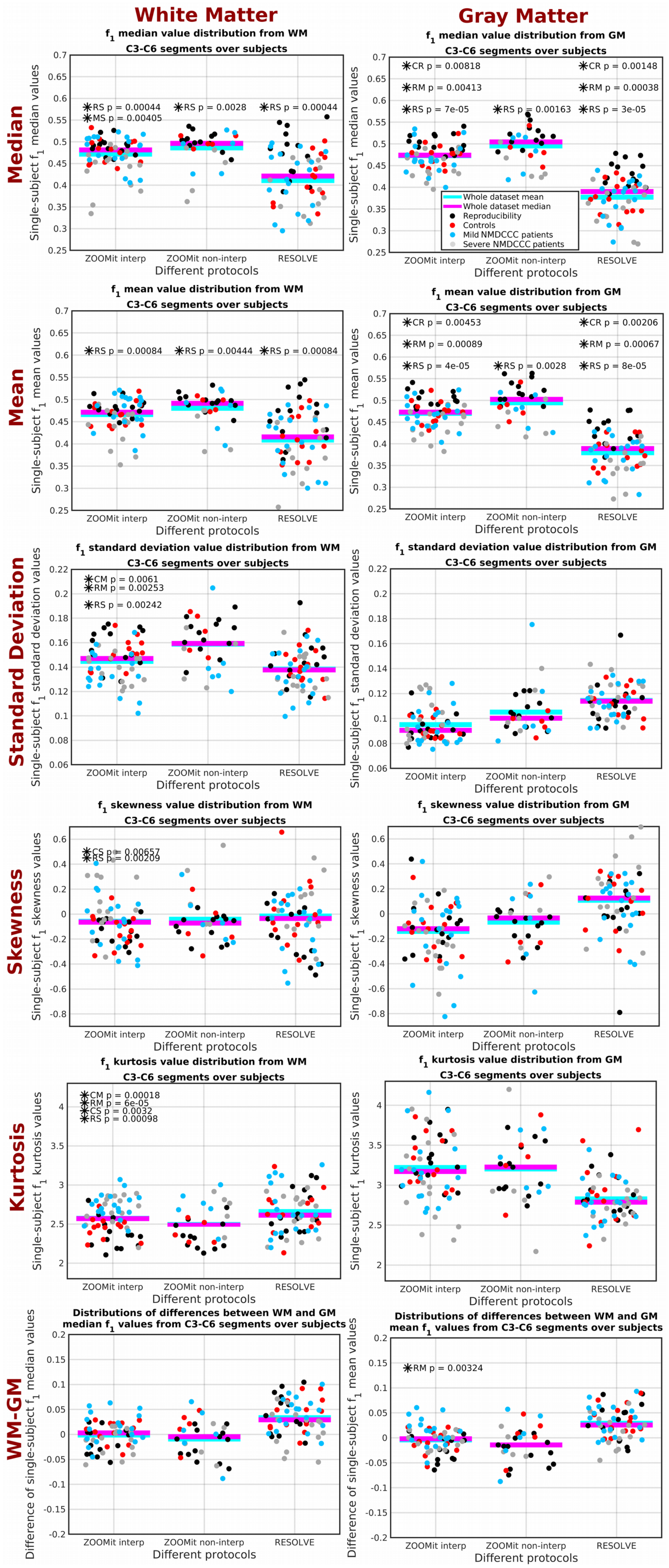

FigS. 2 | Descriptive statistics and WM/GM mean/median gradient results for f_1 maps of three investigated dMRI protocols. Each dot represents a result of a single data acquisition. Study groups are color-coded see caption. P-values are only displayed when the Wilcoxon rank-sum test indicated a significant between-group difference. Group denotation: C - age-comparable control group, R - young healthy volunteers measured twice for protocol reproducibility evaluations, M - patients with mild non-myelopathic degenerative cervical cord compression (NMDCCC), S - patients with severe NMDCCC.

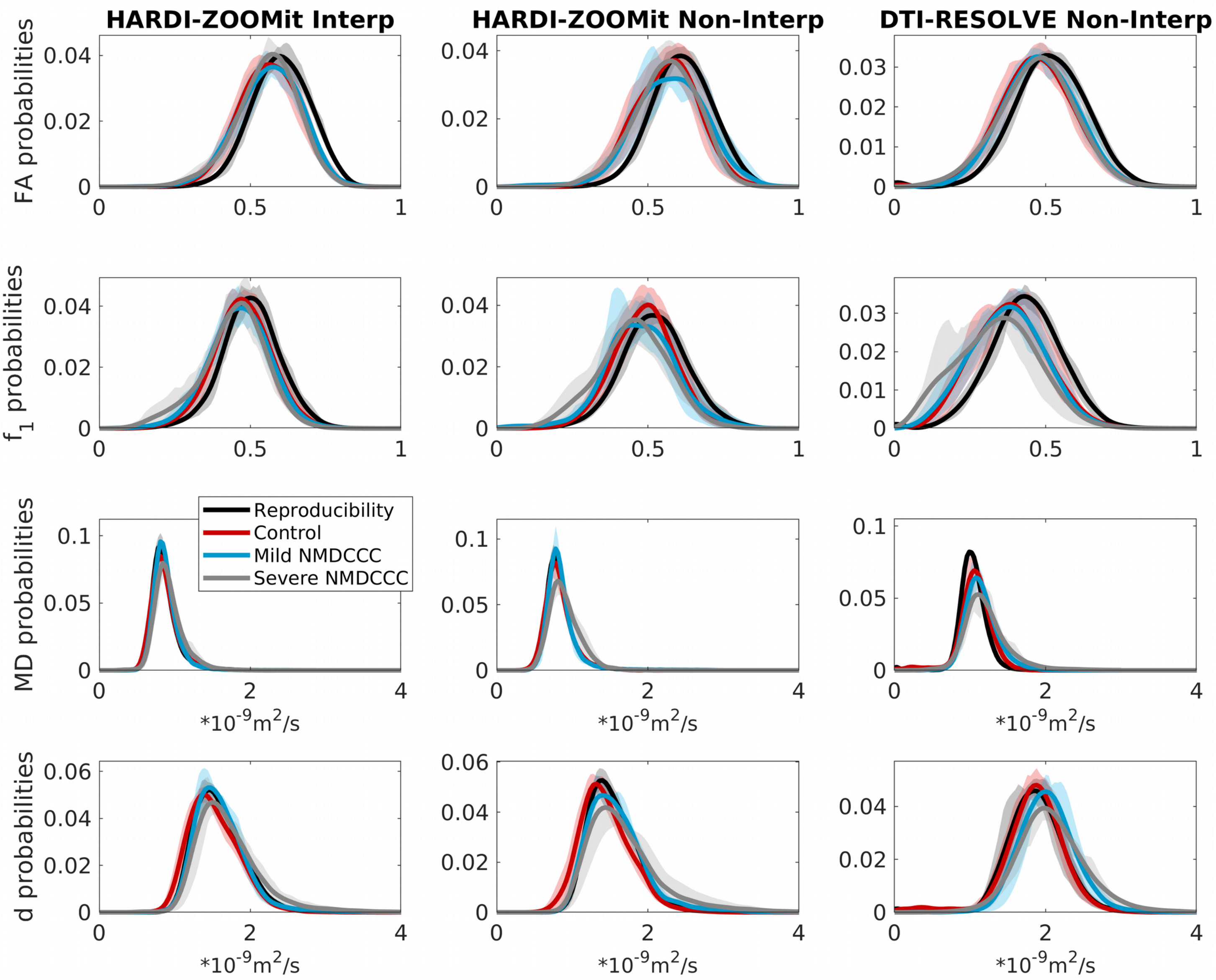

FigS. 3 | Group-averaged kernel-smooth p.d.f.s of dMRI derived microstructural parameters from C3-C6 GM. The confidence intervals (i.e. corresponding color transparent areas) show Q1-Q3 quartiles. Notice that the intervals are narrow and distinct for most of p.d.f.s.

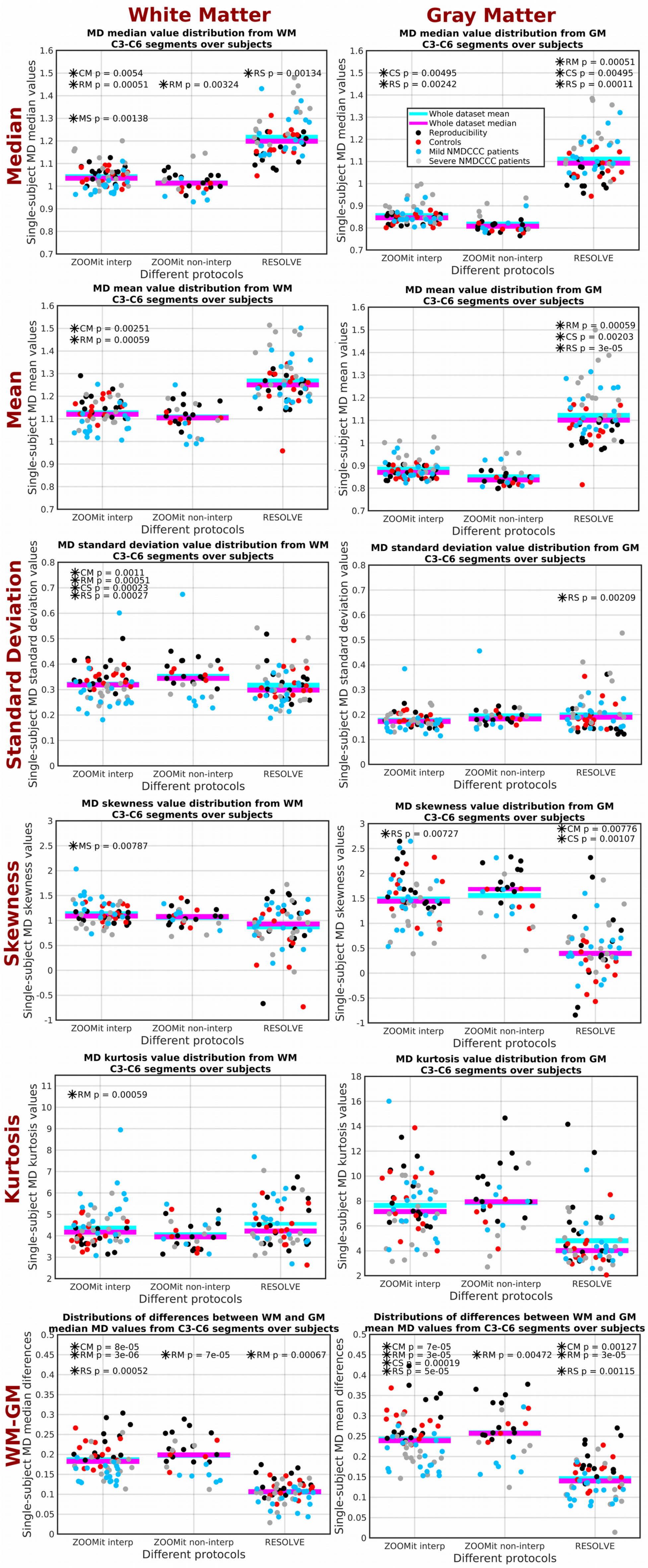

FigS. 4 | Descriptive statistics and WM/GM mean/median gradient results for MD maps of three investigated dMRI protocols. Each dot represents a result of a single data acquisition. Study groups are color-coded see caption. P-values are only displayed when the Wilcoxon rank-sum test indicated a significant between-group difference. Group denotation: C - age-comparable control group, R - young healthy volunteers measured twice for protocol reproducibility evaluations, M - patients with mild non-myelopathic degenerative cervical cord compression (NMDCCC), S - patients with severe NMDCCC.

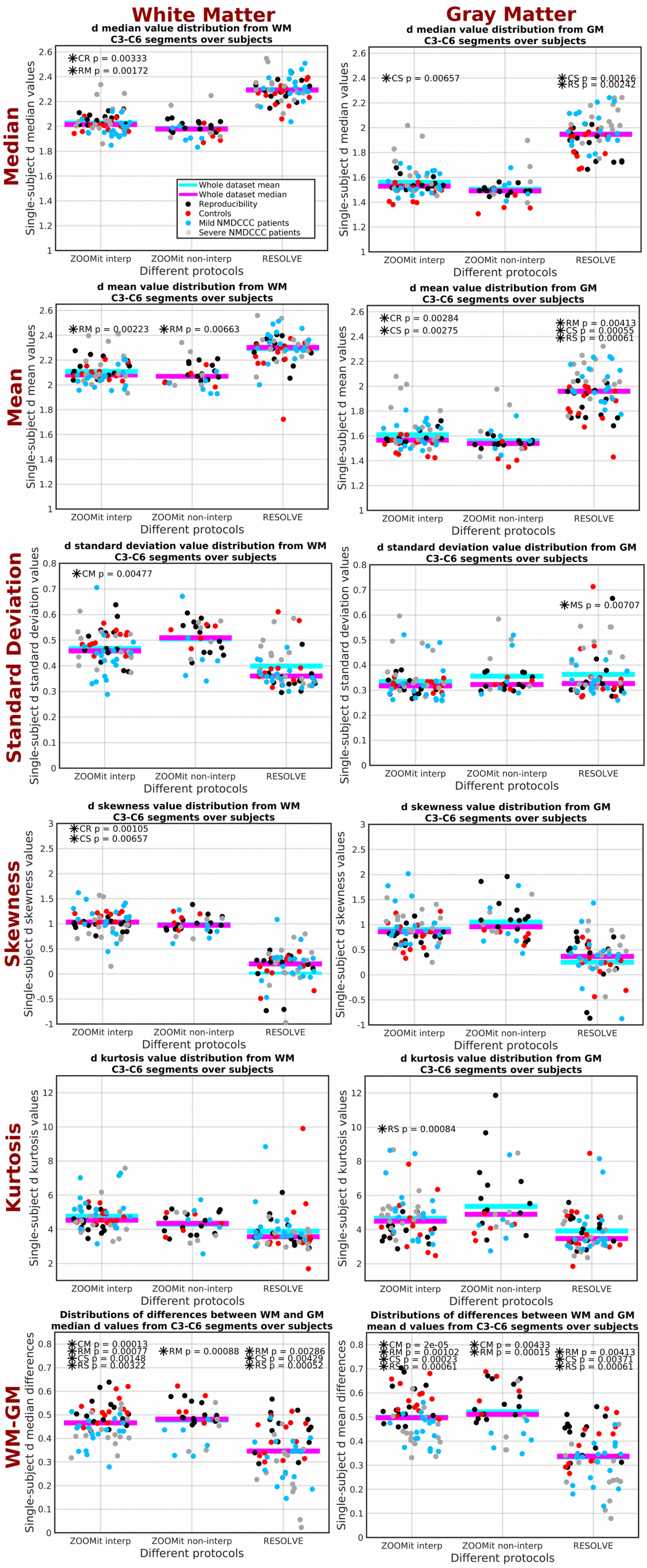

FigS. 5 | Descriptive statistics and WM/GM mean/median gradient results for *d* maps of three investigated dMRI protocols. Each dot represents a result of a single data acquisition. Study groups are color-coded see caption. P-values are only displayed when the Wilcoxon rank-sum test indicated a significant between-group difference. Group denotation: C - age-comparable control group, R - young healthy volunteers measured twice for protocol reproducibility evaluations, M - patients with mild non-myelopathic degenerative cervical cord compression (NMDCCC), S - patients with severe NMDCCC.

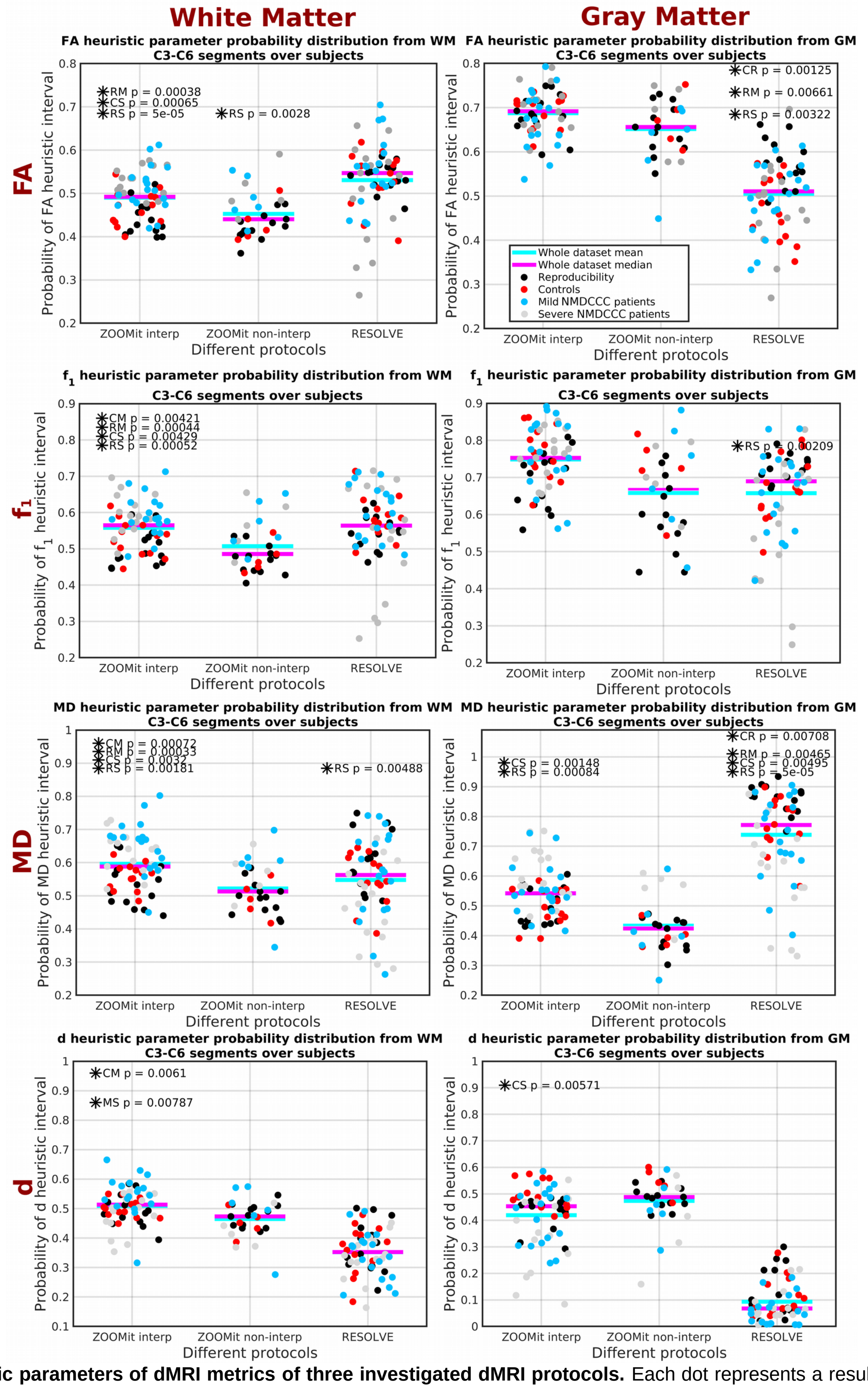

FigS. 6 | Heuristic parameters of dMRI metrics of three investigated dMRI protocols. Each dot represents a result of a single data acquisition. Study groups are color-coded see caption. P-values are only displayed when the Wilcoxon rank-sum test indicated a significant between-group difference. Group denotation: C - age-comparable control group, R - young healthy volunteers measured twice for protocol reproducibility evaluations, M - patients with mild non-myelopathic degenerative cervical cord compression (NMDCCC), S - patients with severe NMDCCC.

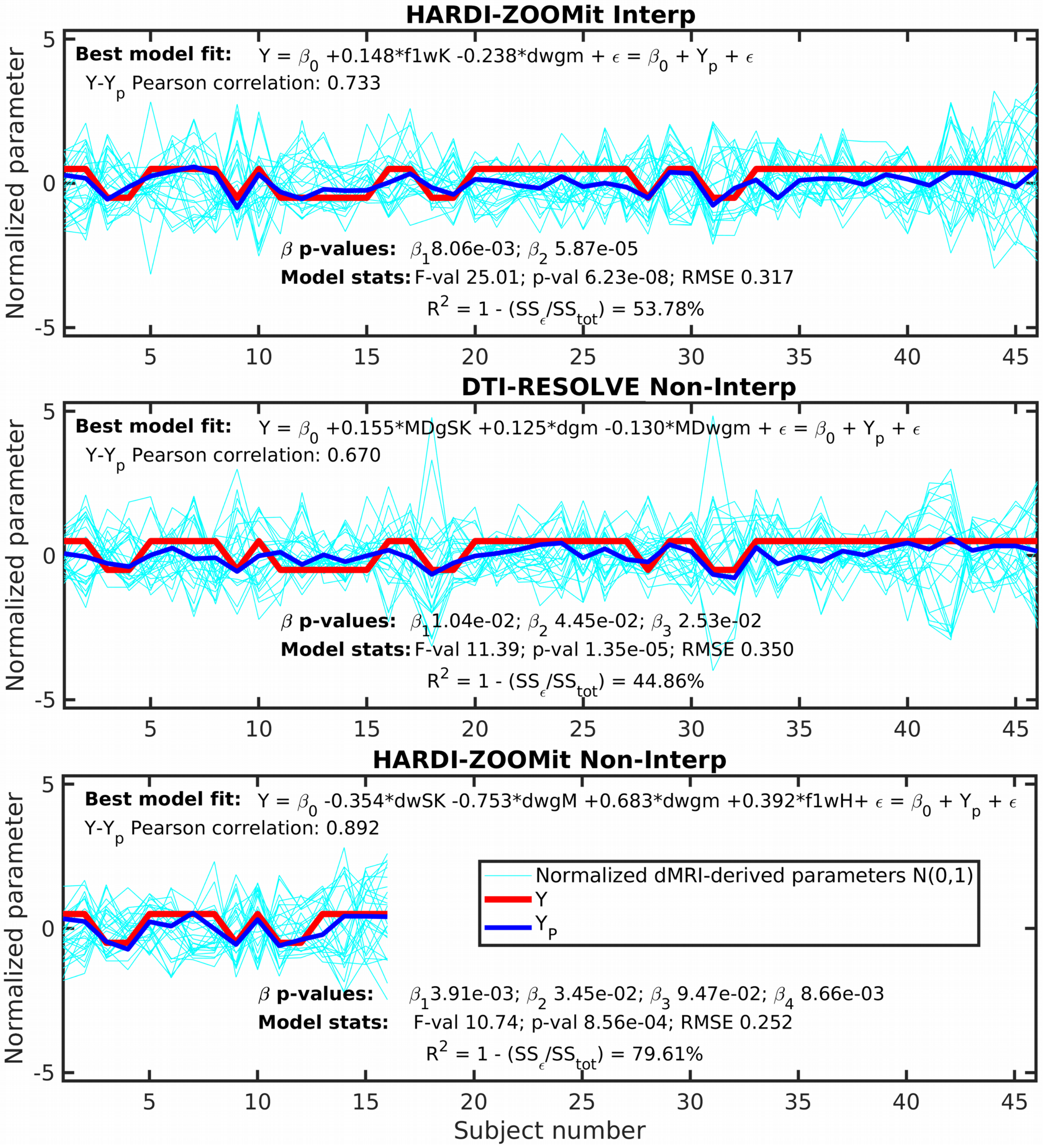

FigS. 7 | Best model fits using step-wise linear regression of dMRI metric parameters that showed significant difference between a group of patients and age-comparable control group for each investigated dMRI protocol. An expected data (Y) was a signal dividing dMRI acquisitions *i* at healthy (Y_i=0.5) and patients (Y_i=-0.5). Best model fit equations with significant variables (i.e. p<0.05 of the variable) and regression coefficients $\beta_{1\text{-}4}$ are shown on the top of each graph. Under the equation, there is a Pearson correlation coefficient between expected (Y) and fitted signal (Y_p). Under signal plots, there are several parameters more characterizing the quality of the expected signal fit from significant dMRI metrics, i.e. P-values of regression coefficients $\beta_{1\text{-}4}$, model fit F-value, model fit p-value, RMSE – root mean square error, and fitted model explained variance R^2.

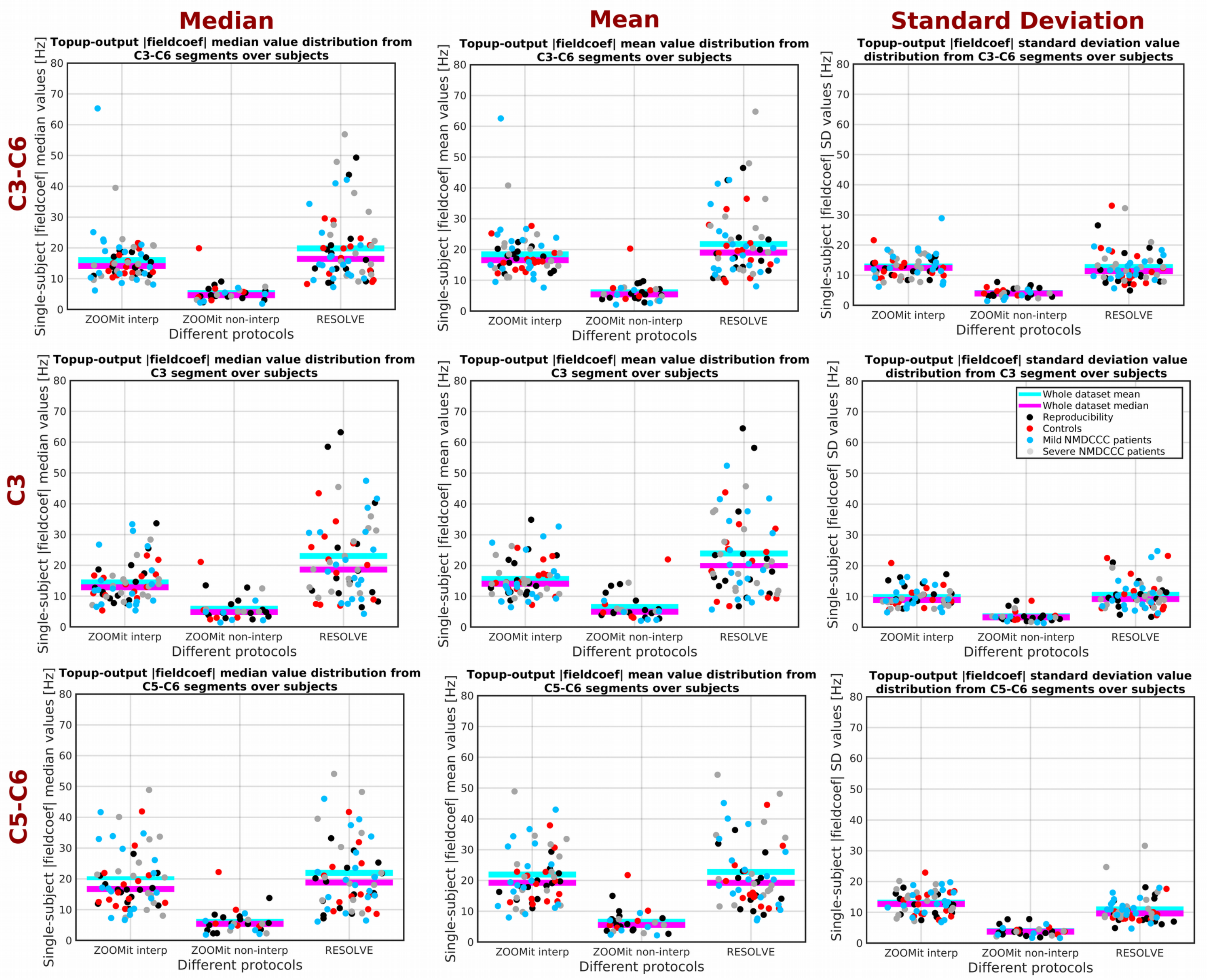

FigS. 8 | Off-resonance effects in three investigated dMRI protocols extracted from 3 different ROIs. Off-resonance effects in between-group comparisons were not significant. |Fieldcoeff| values at y-axis in each graph were estimated from the *"topup"* function implemented in *"fsl"* software library (*fMRIB, Oxford, UK*).
